# Small Area Variation in the Quality of Maternal and Newborn Care in India

**DOI:** 10.1001/jamanetworkopen.2022.42666

**Published:** 2022-11-28

**Authors:** Hwa-Young Lee, Md Juel Rana, Rockli Kim, S. V. Subramanian

**Affiliations:** 1 Department of Global Health and Population, Harvard T.H. Chan School of Public Health, Boston, Massachusetts; 2 Institute of Convergence Science, Convergence Science Academy, Yonsei University, Seoul, South Korea; 3 Korea University Research and Business Foundation, Seoul, South Korea; 4 Division of Health Policy and Management, College of Health Science, Korea University, Seoul, South Korea; 5 Interdisciplinary Program in Precision Public Health, Department of Public Health Sciences, Graduate School of Korea University, Seoul, South Korea; 6 Harvard Center for Population and Development Studies, Cambridge, Massachusetts; 7 Department of Social and Behavioral Sciences, Harvard T.H. Chan School of Public Health, Boston, Massachusetts

## Abstract

**Importance:**

In India, the district serves as the primary policy unit for implementing and allocating resources for various programs aimed at improving key developmental and health indicators. Recent evidence highlights that high-quality care for mothers and newborns is critical to reduce preventable mortality. However, the geographic variation in maternal and newborn health service quality has never been investigated.

**Objective:**

To examine the variation between smaller areas within districts in the quality of maternal and newborn care in India.

**Design, Setting, and Participants:**

This cross-sectional study assessed data from women aged 15 to 49 years on the most recent birth (singleton or multiples) in the 5 years that preceded the fifth National Family Health Survey (June 17, 2019, to April 30, 2021).

**Exposures:**

Maternal and newborn care in 36 states and union territories (UTs), 707 districts, and 28 113 clusters (small areas) in India.

**Main Outcomes and Measures:**

The composite quality score of maternal and newborn care was defined as the proportion of components of care received of the total 11 essential components of antenatal and postnatal care. Four-level logistic and linear regression was used for analyses of individual components of care and composite score, respectively. Precision-weighted prevalence of each component of care and mean composite score across districts as well as their between–small area SD were calculated.

**Results:**

The final analytic sample for the composite score was composed of 123 257 births nested in 28 113 small areas, 707 districts, and 36 states/UTs. For the composite score, 58.3% of the total geographic variance was attributable to small areas, 29.3% to states and UTs, and 12.4% to districts. Of 11 individual components of care, the small areas accounted for the largest proportion of geographic variation for 6 individual components of care (ranging from 42.3% for blood pressure taken to 73.0% for tetanus injection), and the state/UT was the largest contributor for 4 components of care (ranging from 41.7% for being weighed to 52.3% for ultrasound test taken). District-level composite score and prevalence of individual care components and their variation across small areas within the districts showed a consistently strong negative correlation (Spearman rank correlation ρ = −0.981 to −0.886). Low-quality scores and large between–small area disparities were not necessarily concentrated in aspirational districts (mean district composite score [SD within districts], 92.7% [2.1%] among aspirational districts and 93.7% [1.8%] among nonaspirational districts).

**Conclusions and Relevance:**

The findings of this cross-sectional study suggest that the policy around maternal and child health care needs to be designed more precisely to consider district mean and between–small area heterogeneity in India. This study may have implications for other low- and middle-income countries seeking to improve maternal and newborn outcomes, particularly for large countries with geographic heterogeneity.

## Key Points

**Question:**

How much do small areas contribute to the geographic variation in quality of maternal and newborn care in India?

**Findings:**

In this cross-sectional study including 123 257 children, the largest share of geographic variance in maternal and newborn care quality was attributed to small areas in India. The lower the mean composite quality score the districts had, the larger the variation between small areas within the district.

**Meaning:**

These findings highlight the importance of considering heterogeneity within districts to improve maternal and newborn outcomes in India.

## Teaser

This cross-sectional study uses data from fifth National Family Health Survey to measure the multilevel geographic variance, precision-weighted estimates, and small area variation in the quality of maternal and newborn care in India.

## Introduction

Low- and middle-income countries (LMICs) significantly improved maternal and newborn health outcomes during the millennium development goals period by vastly expanding access to health services and basic infrastructure. However, gains in health outcomes have not been proportional to improvement in access. Kruk et al^1^ reported that in 18 countries where 80% to 90% of births were attended by skilled attendants, there are still large gaps in maternal and neonatal mortality. It has been suggested that a portion of this residual mortality can be attributed to poor quality of care.^2^

Quality of care can be categorized into 3 attributes: the structure or inputs to care (material and human resources), the process or component of care (what is actually done), and outcomes of care (effects of care),^3,4^ among which the process attributes are directly related to delivery of care. However, most data sources on health system performance focus exclusively on input metrics, although their correlation with the process of care quality is weak.^5,6^

Most policies and interventions in India are largely designed for and implemented at the district level. One instance is the Transformation of Aspirational Districts initiative launched in 2018. This initiative identified a total of 111 aspirational districts that scored poorly on composite indicators based on key developmental domains, including health, with an aim to accelerate progress in those districts. As a result, quality of health services is one of the indicators monitored for tracking the progress of the initiative. However, factors that affect health service quality operate not only at the district level but also across other geographic levels. Local government capacity is a typical example of contextual factors at the district level. Strong local leadership and active community accountability are community-level contextual variables that have been linked to better-quality health services.^7^ Individual and facility factors associated with service quality, such as patient’s wealth and educational level, are clustered at different levels.^8^ Differential distribution of all these factors across the multiple geographic levels of India may shape geographic variation in health service quality.

Most previous studies^9,10,11^ on geographic variation of health system performance have focused on health outcomes of care, with process quality being only rarely examined in terms of geographic distribution. A few existing studies^12,13^ on between-areas variation in process quality have relied on single-level analysis, such as national or state levels, which may conceal inequalities at lower levels. Previous studies^14,15,16,17,18,19,20,21^ have, however, shed light on small area variation within districts in other indicators, such as undernutrition and low birth weight in India, emphasizing the importance of more precise targeting of interventions.

Under this context, we sought to measure the multilevel geographic variance, precision-weighted estimates, and small area variation in the quality of maternal and newborn care in India. Our specific objectives were as follows: (1) to partition the total geographic variation in composite quality score and prevalence of individual components of maternal and newborn care across 36 states and union territories (UTs), 707 districts, and 28 113 small areas (or clusters); (2) to calculate precision-weighted district mean composite quality scores and prevalence and small area variation of individual components of maternal and newborn care across 707 districts in India; (3) to assess the association between the precision-weighed prevalence of individual components of care and mean composite quality score and their variation between small areas within the district across 707 districts; and (4) on the basis of our findings, to identify the districts that require the highest policy attention to improve the quality of maternal and newborn care.

## Methods

### Data Source and Sample

This project used publicly accessible secondary data obtained from the Demographic and Health Survey (DHS) website. Interviews in DHS were conducted only if the respondent provided voluntary oral informed consent. The deidentified nature of the data does not meet the regulatory definition of human subject research. As such, institutional review board review was not required according to the Harvard Longwood Campus Institutional Review Board. This cross-sectional study followed the Strengthening the Reporting of Observational Studies in Epidemiology (STROBE) reporting guideline.

Data were taken from the fifth National Family Health Survey (NFHS) (June 17, 2019, to April 30, 2021), a nationally representative household survey that covers all 707 districts nested within 29 states and 7 UTs in India.^22^ A stratified 2-stage sampling design was adopted for both urban and rural areas. The primary sampling units—villages, also dubbed as clusters or communities in rural areas and census enumeration blocks in urban areas (hereafter referred to as small areas)—were selected with probability proportional to population size. Households were chosen through systematic random sampling within each primary sampling unit. Further details can be found elsewhere.^22^ We defined the study sample as the most recent birth (singleton or multiples) in the 5 years preceding the survey based on interviews with women aged 15 to 49 years.

### Primary Outcomes

The main variables of interest were quality indicators of maternal and newborn care. To define them, we evaluated maternal reports of antenatal care (ANC) and immediate postnatal care (PNC) components for women who had used the formal health system for each service.^23,24^ We reviewed the World Health Organization guidelines for ANC and PNC to list essential services to be provided by the health system during pregnancy and the postpartum period^25,26^ and then identified corresponding items in the fifth NFHS, comprising 7 components for ANC (weighing pregnant mothers, taking blood pressure, taking a urine sample, taking a blood sample, giving or prescribing iron tablets or syrup, giving tetanus injection, and giving ultrasound testing) and 4 components for PNC (weighing the newborn after birth, examining within 24 hours after birth, a health professional [defined as a physician, nurse, or midwife] examining the newborn, and putting the newborn to breast within 1 hour). Each woman was assigned a composite quality score ranging from 0 to 100, defined as the percentage of components of care received of the total 11 components of care.

We anticipated that the geographic distribution of prevalence of individual care items would vary from one another and from the geographic distribution of the composite score, given their different natures. Specifically, some of the individual care items are actions conducted by the health professional during consultation (such as measuring blood pressure or weighing expectant mothers), which mostly depend on the capacity of the health professional. Other care items have a direct bearing on the availability of infrastructure (eg, ultrasound test and iron prescription). The expertise and experience of health professionals, as well as access to and availability of particular infrastructure, may differ from area to area. We therefore performed our analysis of the prevalence of specific components of care separately from that of the composite quality score.

### Statistical Analysis

We adopted 4-level logistic regression to partition the total variation in the odds of receiving individual components of ANC and PNC, as well as 4-level linear regression models for the composite quality score: the individual unit of inference was defined as a newborn *i* (level 1), and the 3 geographic units of inferences included cluster *j* (or small area, level 2), district *k* (level 3), and state or UT *l* (level 4). We ran 4-level variance component models to decompose the total geographic variation in the probability of a newborn *i* in small area *j*, district *k*, and state/UT *l* receiving specific components of ANC and PNC (eEquation 1 in the Supplement). Based on variance estimates, the 95% population bound (PB) for each level *z* was calculated to assess the range of variability (eEquation 2 in the Supplement). We ran a separate model for variance partitioning of the composite quality score of maternal and newborn care (eEquation 3 in the Supplement). The proportion of the total geographic variance attributable to the level *z* for each of 11 individual components of care and the composite score were estimated by dividing the variance of a given level by the total geographic variation (eEquation 4 in the Supplement). We then conducted a state-stratified 3-level linear regression to estimate the statewide geographic variations for composite quality, where the children are nested in small areas at level 2, which again are nested in districts at level 3.

From the multilevel logistic and linear model, we generated a precision-weighted prevalence of receiving individual components of ANC and PNC (eEquation 5 in the Supplement) and the precision-weighted composite quality score specific to each cluster.^27^ The within-district between-cluster variation was computed as the SD of the prevalence of receiving individual components of ANC and PNC and the SD of the composite quality score. We also generated precision-weighted estimates specific to each district for 11 components of care (eEquation 6 in the Supplement) and their composite score.

We computed the Spearman correlation coefficients (ρ) between the precision-weighted district prevalence of receiving individual components of ANC and PNC and the district mean composite score and the precision-weighted probability and their within-district between-cluster variation (SD). We then identified districts that required high policy attention to improve the quality of maternal and newborn care, defined as those grouped into the bottom 3 deciles of the district mean composite score and the top 3 deciles of between–small areas SD of a composite score. Finally, we divided 707 districts into aspirational and nonaspirational districts using the district code and performed a 2-tailed, paired *t* test to compare the district-level composite score of service quality and small area variation within district. Correlation coefficients and the results from the *t* tests were considered statistically significant at *P* ≤ .05.

## Results

### Sample Characteristics

The final analytic sample for the composite score was composed of 123 257 births nested in 28 113 small areas, 707 districts, and 36 states or UTs (eFigure 1 and eTable 1 in the Supplement). Receiving recommended components of care was reported slightly more frequently for ANC than PNC. The mean (SD) composite quality score was 93.5% (2.8%) (eTable 1 in the Supplement).

### Relative Importance of Geographic Levels

Overall, we found that for the composite score, 58.3% of the total geographic variance was attributable to small areas (ranging from 42.3% for blood pressure taken to 73.0% for tetanus injection), 29.3% to states and UTs, and 12.4% to districts (Table 1). A similar pattern was observed when the analyses were stratified as urban and rural. As for individual components of care, states were the largest source of geographic variation for the following ANC items: being weighed (41.7%), having a blood sample taken (49.3%), having ultrasound test taken (52.3%), and having a urine sample taken (45.7%), with only a small gap between the second-largest contributing level of clusters. For 3 of 4 PNC items, clusters accounted for the largest proportion of the variation, with a significant gap between cluster and states: 57.0% vs 27.4% for being weighed at birth, 65.2% vs 19.5% for being put to breast at 1 hour or earlier, and 58.6% vs 18.4% for being checked by a health professional. Districts were the largest source of variation for only 1 PNC item: checked within 24 hours (57.2%). Districts were the least contributors to the geographic variation for the remaining 10 items (from 11.1% for iron taken or bought to 23.0% for being checked by a health professional) (Table 1). When the variation attributable to the districts and clusters in the composite score was partitioned across India’s 26 states and UTs having more than 5 districts, only 5 states displayed 50% or less of the variation at cluster level (eFigure 2 in the Supplement).

**Table 1.** Four-Level Variance Component Model for Individual Components and Composite Quality Score of Maternal and Newborn Care in India

Score	State	District	Cluster
**Composite score**			
Overall			
Variance estimate (SE)	5.58 (1.50)	2.37 (0.17)	11.12 (0.26)
VPC, %^a^	29.3	12.4	58.3
Mean (95% PB)	94.1 (89.5-98.8)	94.1 (91.1-97.2)	94.1 (87.6-100)
Urban			
Variance estimate (SE)	3.57 (0.97)	1.92 (0.26)	7.49 (0.44)
VPC, %^a^	27.5	14.8	57.7
Mean (95% PB)	94.1 (90.4-97.9)	94.1 (91.4-96.9)	94.1 (88.8-99.5)
Rural			
Variance estimate (SE)	5.50 (1.44)	2.62 (0.21)	12.07 (0.32)
VPC, %^a^	27.2	13.0	59.8
Mean (95% PB)	94.4 (89.6-98.7)	94.4 (91.0-97.3)	94.4 (87.3-100)
**Antenatal care**			
Being weighed at birth			
Variance estimate (SE)	1.58 (0.51)	0.69 (0.08)	1.51 (0.08)
VPC, %^a^	41.7	18.3	40.0
Prevalence, mean (95% PB), %	99.6 (95.9-100)	99.6 (98.2-100)	99.6 (96.1-100)
Blood pressure taken			
Variance estimate (SE)	1.22 (0.38)	0.59 (0.06)	1.33 (0.08)
VPC, %^a^	38.9	18.8	42.3
Prevalence, mean (95% PB), %	99.5 (95.9-99.9)	99.5 (97.8-99.9)	99.5 (95.5-99.9)
Blood sample taken			
Variance estimate (SE)	2.05 (0.61)	0.56 (0.05)	1.56 (0.06)
VPC, %^a^	49.3	13.4	37.4
Prevalence, mean (95% PB), %	99.3 (89.3-100)	99.3 (97.0-99.8)	99.3 (92.3-99.9)
Ultrasound test taken			
Variance estimate (SE)	1.64 (0.44)	0.59 (0.04)	0.90 (0.03)
VPC, %^a^	52.3	18.9	28.8
Prevalence, mean (95% PB), %	95.2 (61.7-99.6)	95.2 (81.4-98.9)	95.2 (75.5-99.2)
Urine sample taken			
Variance estimate (SE)	1.90 (0.54)	0.58 (0.05)	1.68 (0.07)
VPC, %^a^	45.7	14.0	40.3
Prevalence, mean (95% PB), %	99.1 (88.1-99.9)	99.1 (96.1-99.8)	99.1 (89.7-99.9)
Tetanus injection taken			
Variance estimate (SE)	0.18 (0.06)	0.23 (0.03)	1.12 (0.07)
VPC, %^a^	12.0	15.0	73.0
Prevalence, mean (95% PB), %	97.8 (95.1-99.0)	97.8 (94.6-99.1)	97.8 (84.9-99.7)
Iron taken or bought			
Variance estimate (SE)	0.90 (0.26)	0.23 (0.02)	0.93 (0.04)
VPC, %^a^	43.6	11.1	45.4
Prevalence, mean (95% PB), %	95.5 (76.8-99.3)	95.5 (89.3-98.2)	95.5 (76.2-99.3)
**Postnatal care**			
Being weighed at birth			
Variance estimate (SE)	0.46 (0.15)	0.26 (0.04)	0.96 (0.09)
VPC, %^a^	27.4	15.6	57.0
Prevalence, mean (95% PB), %	99.3 (97.5-99.8)	99.3 (98.2-99.8)	99.3 (95.6-99.9)
Put to breast ≤1 h			
Variance estimate (SE)	0.26 (0.07)	0.20 (0.02)	0.87 (0.03)
VPC, %^a^	19.5	15.3	65.2
Prevalence, mean (95% PB), %	84.2 (66.2-93.5)	84.2 (68.8-92.8)	84.2 (46.2-97.1)
Checked by health professionals			
Variance estimate (SE)	0.39 (0.13)	0.49 (0.06)	1.24 (0.12)
VPC, %^a^	18.4	23.0	58.6
Prevalence, mean (95% PB), %	99.5 (98.4-99.9)	99.5 (98.1-99.9)	99.5 (95.8-99.9)
Checked at ≤24 h			
Variance estimate (SE)	0.28 (0.09)	0.40 (0.03)	0.02 (0.01)
VPC, %^a^	39.9	57.2	2.9
Prevalence, mean (95% PB), %	96.7 (91.2-98.8)	96.7 (89.5-99.0)	96.7 (95.7-97.5)

Abbreviations: PB, population bound; VPC, variance partitioning coefficient.

^a^ VPC is based on geographic variance, excluding individual-level variance.

### Within-District Small Area Variation in the Quality of Maternal and Newborn Care

**Figure 1.**
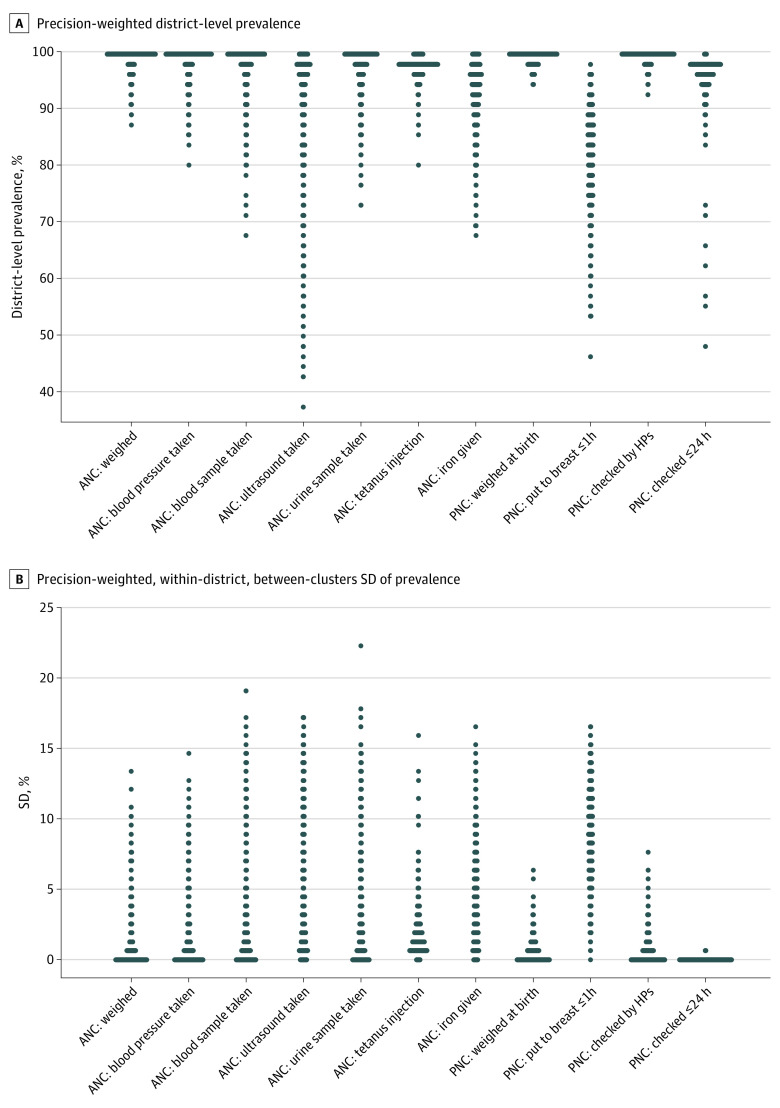
Prevalence of Individual Components of Care Received Each dot represents 1 district, with bars representing an overlap of multiple dots; thus, a wide bar indicates a high frequency of values. ANC indicates antenatal care; HP, health professional; PNC, postnatal care.

The district-level composite score and prevalence of individual service items were generally high, and substantial variation was found among different districts. The precision-weighted district mean composite score ranged from 82.8% to 98.4% (mean [SD], 93.5% [2.8%]). The district prevalence of ultrasound test taken ranged from 37.8%-99.7%, and the prevalence of newborn being put to breast within an hour ranged from 47.0% to 97.6%, with an overall mean (SD) prevalence as high as 89.2% (5.0%) for ultrasound test taken and 81.6% (8.7%) for being put to breast within an hour (eTable 2 in the Supplement; Figure 1A). Within-district, the mean (SD) between–small area SD was noticeably high for being put to breast within 1 hour (8.7%) but was lower than 1 for the other 3 PNC items (eTable 2 in the Supplement; Figure 1B).

**Figure 2.**
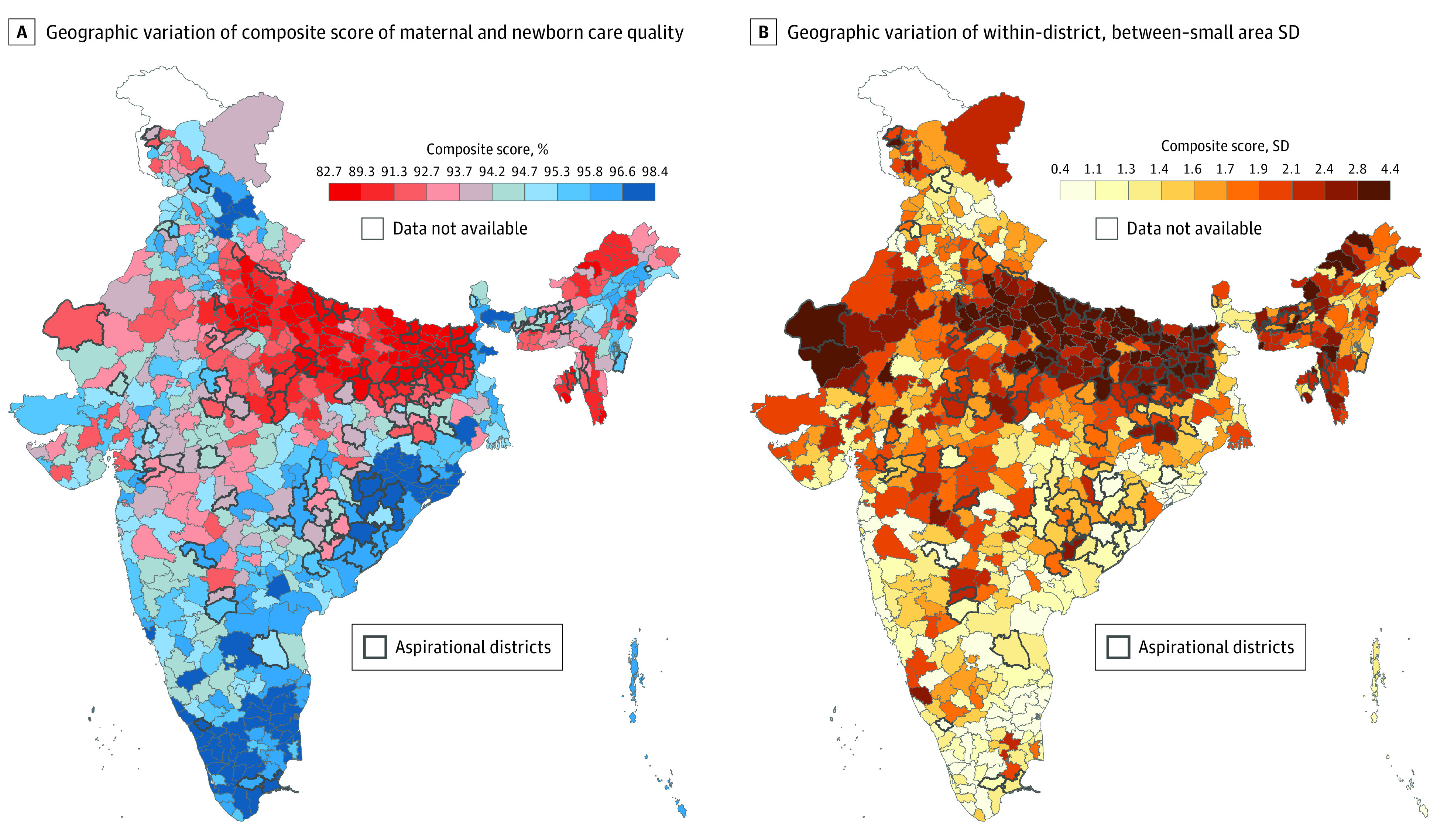
Geographic Variation of Composite Scores of Maternal and Newborn Care Quality Across 707 Districts in India

Districts with poor quality of maternal and newborn care and high within-district variation were concentrated in the northern states of India: Rajasthan, Uttar Pradesh, and Bihar (Figure 2). Districts in the southern part of the country, especially in the states of Karnataka, Odisha, Tamil Nadu, and Andhra Pradesh, were mostly grouped into the highest or the second highest deciles of the composite score and the lowest decile of between–small area variation (Figure 2).

The spatial distribution of the district prevalence and between–small area variation displayed a different pattern depending on the individual component of care. For instance, although districts with a low prevalence and high between–small areas inequality of newborns being weighed at birth were heavily concentrated in northern Indian states, districts with a low prevalence and high between–small area SD of tetanus injection were dispersed widely rather than concentrated in specific states (eFigure 3A and B in the Supplement).

### Correlation Between District Mean and Small Area Variation in the Quality of Maternal and Newborn Care

**Figure 3.**
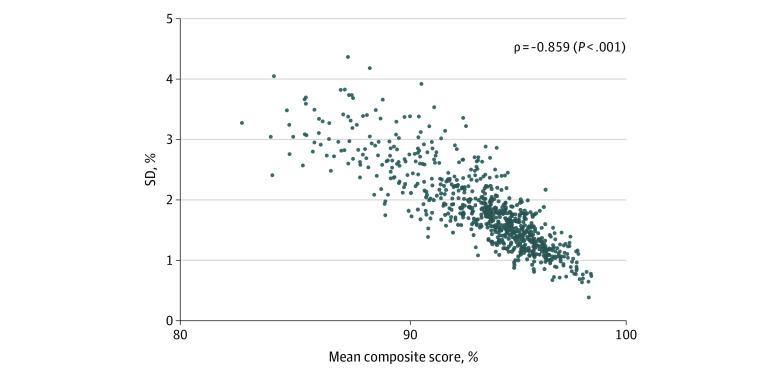
Correlation Between District-Level Composite Quality Score and Within-District, Between-Clusters SD of Composite Score of Maternal and Newborn Care Quality Across 707 Districts in India

Spearman correlation showed a strong negative correlation between district mean composite score and between–small area SD (ρ = −0.859, *P* < .001) (Figure 3). eFigure 4 in the Supplement shows a consistently strong negative correlation between district prevalence and within-district between–small area SD for all 11 individual components of care (ρ = −0.981 to −0.886, *P* < .001 for all), indicating that districts where the quality of maternal and newborn care is low have higher between–small area inequality.

### Quality of Maternal and Newborn Care in Aspirational Districts

The mean composite score was slightly lower and the SD in the score between small areas within the district was slightly higher among aspirational districts than among nonaspirational districts, with statistically significant differences (mean [SD], 92.7% [2.1%] vs 93.7% [1.8%]; *P* < .001) (Table 2). Based on our results, we identified 166 districts that required high policy attention for having high overall burden and substantial inequality (defined as aforementioned) (eTable 3 in the Supplement). Only 42 of the 111 aspirational districts overlapped with these districts.

**Table 2.** Mean of District-Level Composite Score and Between-Clusters SD of Aspirational and Nonaspirational Districts

	Aspirational district		*P* value
	Yes (n = 111)	No (n = 596)	
District composite score, %			
Mean	92.7	93.7	<.001
Median (range)	93.3 (84.8-97.6)	94.3 (82.8-98.4)	
Within-district, between-clusters SD, %			
Mean	2.1	1.8	<.001
Median (range)	2.0 (0.9-4.4)	1.7 (0.4-4.4)	

## Discussion

Based on 11 components of ANC and PNC, we aimed to quantify the variation in quality of care for pregnant mothers and newborns across 4 policy-relevant geographic levels in India. Four salient findings emerged from our analyses. First, of the multiple geographic locations evaluated, small area (or cluster) was the largest source of variation in the composite quality score, whereas the contribution of districts was the least significant. Of 11 individual care components, the state was the largest source of geographic variation for 4 ANC components, but by only a small difference in the variance partition coefficient from small area. Small area was the largest source of geographic variation for 3 ANC and 3 PNC components, showing a significant gap with the state. Second, the district prevalence of 11 individual components of care, the district mean composite score, and the within-district, between–small area SD had wide ranges across 707 districts. Overall, maternal and newborn care quality was lower in districts, and between–small area variation within districts was greater in northern parts of India than in other areas. Spatial distribution of district-level prevalence and small area SD of some individual components of care showed different patterns from district-level composite score distribution and its small area SD. Third, the correlation between the district composite score of quality of care and prevalence of individual care components and within-district, between–small area SD was negative, implying that the districts with lower-quality care also tend to have a higher degree of between–small area inequality. Fourth, although aspirational districts were lower in overall district composite score and higher in between–small area SD compared with nonaspirational districts, the gap was minimal; districts identified as requiring high policy attention for improving the quality of maternal and newborn care overlapped with only one-third of aspirational districts.

Our study has several policy implications. First, India is a populous country with huge heterogeneities in terms of local government capacity, socioeconomic status, culture, and other factors. As a result, identifying the most relevant policy units is crucial for designing policy that makes the most efficient use of limited resources. In India, all major underlying indicators for development policies and interventions are available at the administrative units of the district (eg, the District Level Household & Facility Survey and Health Management Information System).^28,29^ Resource allocation is also currently decided at the district level, without further taking into account variation among smaller areas within each district. Our findings, in contrast, do not support this current policy approach. We found that the greatest proportion of variation in the quality of maternal and newborn care lies at the small area level, whereas the least variation was at the between-district level. Health professionals administering competent care may be concentrated in affluent villages. Even when the quality of health professionals is mixed, better-educated mothers may be able to identify and choose competent health professionals, whereas mothers with low educational levels may not be able to do so.^30^ These socioeconomic status characteristics tend to cluster more densely at the household or small area level than at the higher areas, which may be one of the mechanisms causing greater variation among small areas.

Our findings underscore the statistical importance of small areas for effectively implementing, monitoring, overseeing, and evaluating maternal and newborn care policies, suggesting that the current district-focused approach should be revised. For example, the Barmer district in Rajasthan had a relatively high district mean composite quality score for maternal and newborn care (eTable 3 in the Supplement). Nonetheless, it was grouped into the highest decile for between–small area variation, indicating the coexistence of small areas offering both extremely high and low quality of care. By relying only on district means, small areas with poor quality scores nested within districts with high mean quality scores may be overlooked. The National Health Mission is one of the Indian government’s initiatives whose approach aligns with our findings. The focus of the mission is on establishing a community-owned, decentralized health delivery system with intersectoral convergence at all levels, highlighting the importance of bolstering its community-based components, such as enhancing the role of the Panchayati Raj Institution (a system of local self-government of villages).^31^

Second, a different spatial distribution of individual components of care from the overall composite score emphasizes the need to examine each care component separately in addition to the composite score. Deciding which components of maternal and newborn care should be prioritized for improvement should depend on the profile of each small area.

Third, a negative correlation between district composite scores and between–small area SD is in line with the results from prior studies in which districts with a high prevalence of unfavorable nutritional status (ie, undernutrition and low birth weight) show larger inequality among small areas.^14,21^ Therefore, we argue that differentiating resource allocation across small areas even within the aspirational districts needs to be considered.

Fourth, the composite service quality score for maternal and newborn care and its between–small area variation only slightly differs between aspirational and nonaspirational districts. Maternal and child health is one of the subjects that the aspirational district program aims to tackle to meet the sustainable development goal targets on maternal and neonatal mortality. A few of the indicators used to select and closely monitor aspirational districts, such as the percentage of newborns breastfed within 1 hour and the percentage of live newborns weighed at birth, overlapped with the individual care components used in our analyses. However, improving maternal and newborn health outcomes can be achieved by a continuum of comprehensive ANC and PNC throughout pregnancy, labor, and the postpartum period, rather than practicing only a few specific care actions. Thus, the adjustment of aspirational districts based on a broader range of service quality metrics needs to be considered. Districts identified in our study as having low composite quality scores and high between–small area variation should be prioritized in addition to aspirational districts to achieve meaningful gains in maternal and child health outcomes and reduce geographic inequality at the same time.

### Limitations

This study has a few limitations. First, the components of care used to create a quality score for maternal and newborn care were self-reported by mothers and may be subject to error. However, recent evidence has shown that women in 3 LMICs (Bangladesh, Cambodia, and Kenya) were able to recall routine interventions they received during the ANC and PNC periods with accuracy, regardless of educational level. The validity was higher for indicators related to concrete, observable actions (eg, examination or medication taken) rather than for information or advice given during counseling (eg, discussion of HIV).^32^ Given that all the individual care items in our analyses correspond to the former and were likely recognizable to the respondents, any significant bias resulting from self-report is not anticipated to arise. Second, although some ANC components (eg, weight assessment and blood pressure measurement) should be completed in all ANC visits, the NFHS only examined whether mothers received a particular care item throughout the entire pregnancy. However, other ANC components (eg, ultrasound screening and iron prescriptions) are not anticipated to be provided at every visit. In addition, the PNC components we used in our analysis address whether health professionals met certain criteria in providing the service rather than whether they offered a particular care item. Thus, although our composite indicator is not entirely comprehensive, we assume that its soundness has not been undermined, which is further supported by a prior study^33^ showing a significant association among quintiles of district mean composite score constructed in the same manner as ours and neonatal and early neonatal mortality saved by institutional delivery compared with home birth. Third, although villages in rural areas and blocks in urban areas were defined as small areas, these areas are not directly equivalent in terms of size and administrative role.

## Conclusions

This cross-sectional study has demonstrated that health service quality for maternal and newborn care in India is highly variable not only across districts but also across small areas within any given district. These findings suggest that health programs and interventions that target pregnant mothers and newborns should consider a more precise design based on within-district small area variation in addition to district-level means. More specific interventions may be designed by identifying small area features associated with local quality of maternal and newborn care in future research. This study may have implications for other LMICs seeking to improve maternal and newborn outcomes, particularly for large countries with geographic heterogeneity.

## Notes

Supplement.
eEquation 1.

eEquation 2.

eEquation 3.

eEquation 4.

eEquation 5.

eEquation 6.
**eFigure 1.** Schematic of Analytic Sample Selection Procedure With Hierarchy of Sampling Distribution for Composite Quality Score of Maternal and Newborn Care in India, NFHS-5 (2019-2020) **eFigure 2.** Geographic Variation of Average Composite Score for Maternal and Newborn Care Quality Attributable to the Cluster (%) Across the States and Union Territories of India, NFHS-5 (2019-2020) (States With 5 or Less Districts Were Not Included Due to the Reliability of Estimates) **eFigure 3.** Geographic Variation of Prevalence and Within-District, Between-Clusters Standard Deviation of Individual Care Contents Received Across 707 Districts, India **eFigure 4.** Correlation Between Prevalence of Individual Care Contents Received and Within-District, Between-Clusters SD of Them Across 707 Districts in India **eTable 1.** Contents of Antenatal Care (ANC) and Postnatal Care (PNC) to Construct the Composite Score for Maternal and Newborn Care Quality **eTable 2.** District-Level Precision-Weighted Prevalence of Individual Contents of Care Received and Composite Quality Score and Standard Deviation (SD) of Them Between Clusters **eTable 3.** District-Level Average Composite Score and Within-District, Between-Clusters SD of Them and Their Positions in the Decile Distribution, India, 2019-2020
