# Supplementary material for: Small Area Variation in the Quality of Maternal and Newborn Care in India
Source: JAMA Netw Open. 2022 Nov 28;5(11):e2242666. doi: 10.1001/jamanetworkopen.2022.42666 (PMC9706367; doi:10.1001/jamanetworkopen.2022.42666)
Supplement: Supplement. — eEquation 1. eEquation 2. eEquation 3. eEquation 4. eEquation 5. eEquation 6. eFigure 1. Schematic of Analytic Sample Selection Procedure With Hierarchy of Sampling Distribution for Composite Quality Score of Maternal and Newborn Care in India, NFHS-5 (2019-2020) eFigure 2. Geographic Variation of Average Composite Score for Maternal and Newborn Care Quality Attributable to the Cluster (%) Across the States and Union Territories of India, NFHS-5 (2019-2020) (States With 5 or Less Districts Were Not Included Due to the Reliability of Estimates) eFigure 3. Geographic Variation of Prevalence and Within-District, Between-Clusters Standard Deviation of Individual Care Contents Received Across 707 Districts, India eFigure 4. Correlation Between Prevalence of Individual Care Contents Received and Within-District, Between-Clusters SD of Them Across 707 Districts in India eTable 1. Contents of Antenatal Care (ANC) and Postnatal Care (PNC) to Construct the Composite Score for Maternal and Newborn Care Quality eTable 2. District-Level Precision-Weighted Prevalence of Individual Contents of Care Received and Composite Quality Score and Standard Deviation (SD) of Them Between Clusters eTable 3. District-Level Average Composite Score and Within-District, Between-Clusters SD of Them and Their Positions in the Decile Distribution, India, 2019-2020 [file jamanetwopen-e2242666-s001.pdf]

Supplementary Online Content

Lee H-Y, Rana MJ, Kim R, Subramanian SV. Small area variation in the quality of maternal and newborn care in India. *JAMA Netw Open*. 2022;5(11):e2242666.
doi:10.1001/jamanetworkopen.2022.42666

eEquation 1.
eEquation 2.
eEquation 3.
eEquation 4.
eEquation 5.
eEquation 6.
eFigure 1. Schematic of Analytic Sample Selection Procedure With Hierarchy of Sampling Distribution for Composite Quality Score of Maternal and Newborn Care in India, NFHS-5 (2019-2020)
eFigure 2. Geographic Variation of Average Composite Score for Maternal and Newborn Care Quality Attributable to the Cluster (%) Across the States and Union Territories of India, NFHS-5 (2019-2020) (States With 5 or Less Districts Were Not Included Due to the Reliability of Estimates)
eFigure 3. Geographic Variation of Prevalence and Within-District, Between-Clusters Standard Deviation of Individual Care Contents Received Across 707 Districts, India
eFigure 4. Correlation Between Prevalence of Individual Care Contents Received and Within-District, Between-Clusters SD of Them Across 707 Districts in India
eTable 1. Contents of Antenatal Care (ANC) and Postnatal Care (PNC) to Construct the Composite Score for Maternal and Newborn Care Quality
eTable 2. District-Level Precision-Weighted Prevalence of Individual Contents of Care Received and Composite Quality Score and Standard Deviation (SD) of Them Between Clusters
eTable 3. District-Level Average Composite Score and Within-District, Between-Clusters SD of Them and Their Positions in the Decile Distribution, India, 2019-2020

This supplementary material has been provided by the authors to give readers additional information about their work.

© 2022 Lee H-Y et al. *JAMA Network Open.*

eEquation 1

$logit\ (Pr\ (Y_{ijkl} = 1)) = \beta_0 + (u_{0jkl} + v_{0kl} + f_{0l})$.

Where β_0 represents the constant, while u_{0jkl}, v_{0kl}, and f_{0l} are residuals specific to a small area, district, and state/UT. Each set of residuals are assumed to be normally distributed with a mean of 0 and a variance of $u_{0jkl} \sim N(0, \sigma^2_{u0})$, $v_{0kl} \sim N(0, \sigma^2_{v0})$, and $f_{0l} \sim N(0, \sigma^2_{f0})$. The terms σ^2_{u0}, σ^2_{v0}, and σ^2_{f0} denote variance between-clusters within a district, variance between-districts within a state, and between-states within a country, respectively.

eEquation 2.

95% coverage boundary for level z= $\exp\left(\beta_0 \pm 1.96\sqrt{\sigma^2_z}\right) / (1 + \exp\left(\beta_0 \pm 1.96\sqrt{\sigma^2_z}\right))$

eEquation 3.

$C_{ijkl} = \beta_0 + (e_{0ijkl} + u_{0jkl} + v_{0kl} + f_{0l})$.

eEquation 4.

Proportion of the total geographic variance attributable to the level z = $\left(\frac{\sigma^2_z}{\sigma^2_{u0} + \sigma^2_{v0} + \sigma^2_{f0}} \right) \times 100$.

eEquation 5.

The probability of each child in the clusters receiving specific component of ANC or PNC=

$$\frac{\exp\left[\beta_0 + \left(u_{0jkl} + v_{0kl} + f_{0l}\right)\right]}{1 + \exp\left[\beta_0 + \left(u_{0jkl} + v_{0kl} + f_{0l}\right)\right]}$$

eEquation 6.

The prevalence of receiving specific component of ANC and PNC in the district =

$$\frac{\exp\left[\beta_0 + (v_{0kl} + f_{0l})\right]}{1 + \exp\left(\beta_0 + (v_{0kl} + f_{0l}\right)}$$

© 2022 Lee H-Y et al. *JAMA Network Open.*

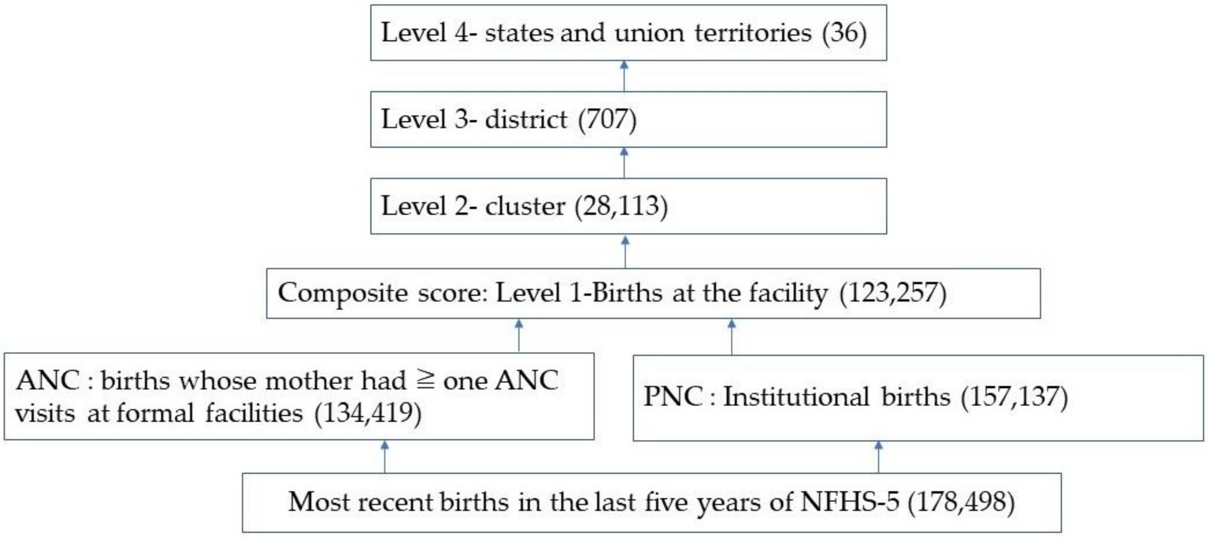

eFigure 1. Schematic of analytic sample selection procedure with hierarchy of sampling distribution for composite quality score of maternal and newborn care in India, NFHS-5 (2019-2020). Sample sizes for estimates of individual contents of ANC and PNC are different due to different inclusion criteria for ANC and PNC quality estimates

© 2022 Lee H-Y et al. *JAMA Network Open.*

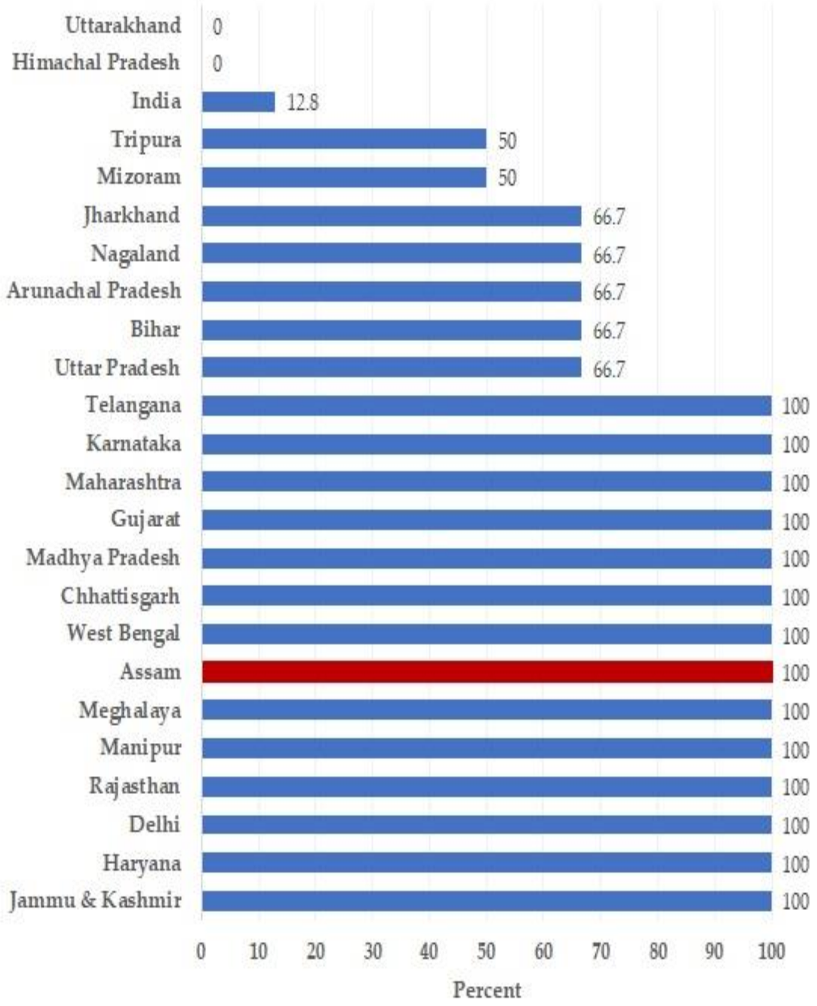

eFigure 2. Geographic variation of average composite score for maternal and newborn care quality attributable to the cluster (%) across the states and union territories of India, NFHS-5 (2019-2020). (States with 5 or less districts were not included due to the reliability of estimates)

© 2022 Lee H-Y et al. *JAMA Network Open.*

eFigure 3. Geographic variation of prevalence and within—district, between-clusters standard deviation of individual care contents received across 707 districts, India.

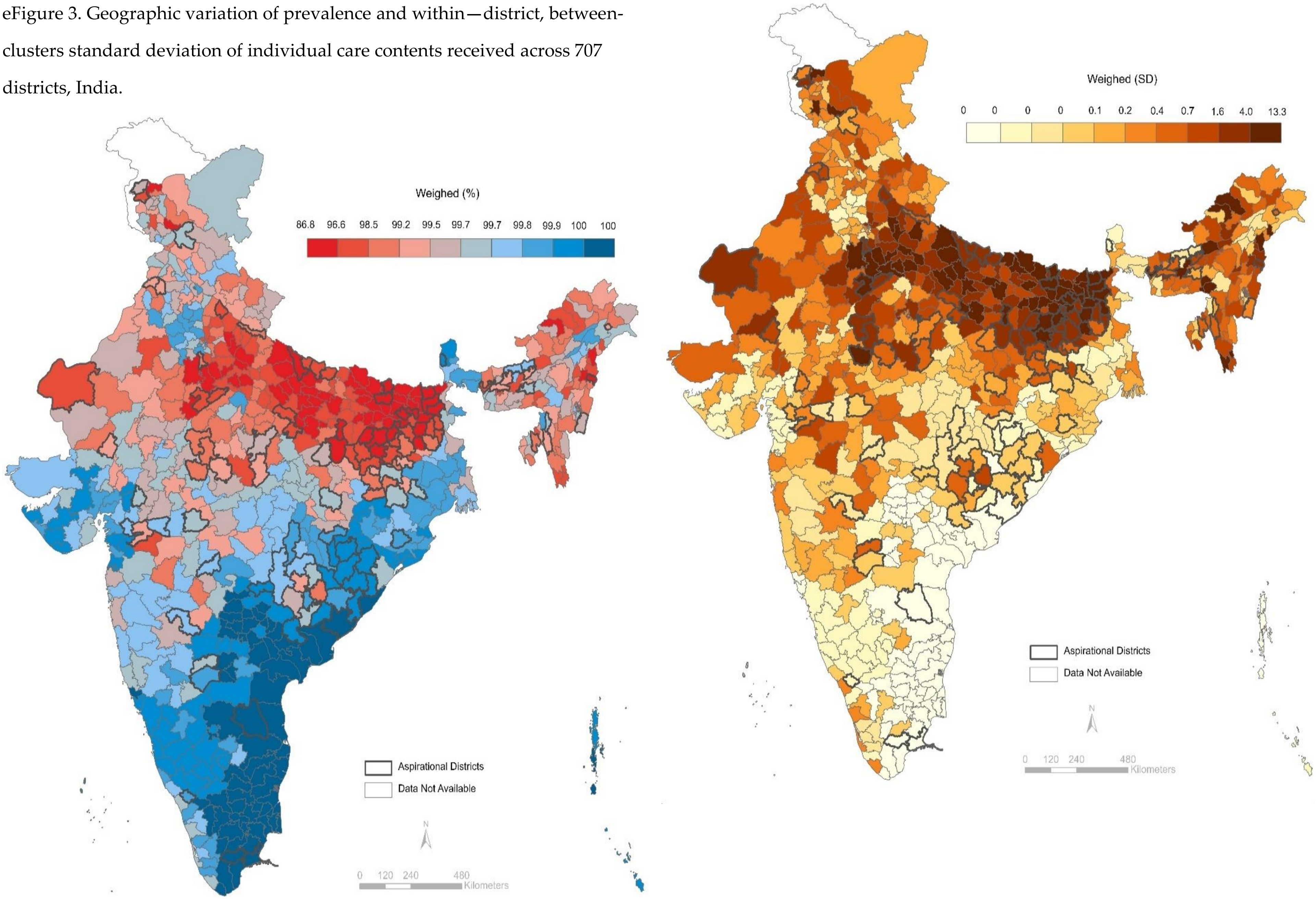

ANC: weighed

© 2022 Lee H-Y et al. *JAMA Network Open.*

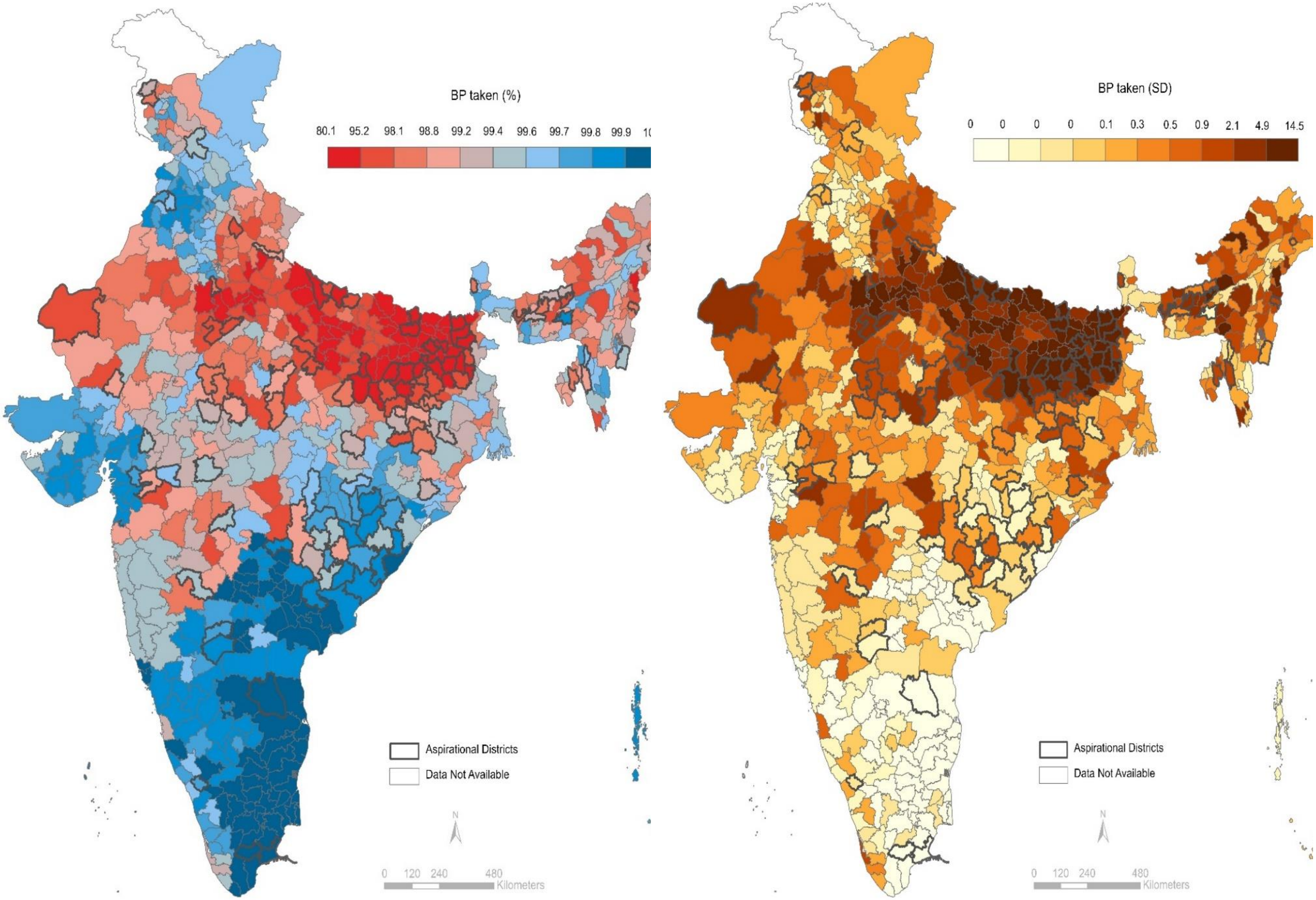

ANC: blood pressure taken

© 2022 Lee H-Y et al. *JAMA Network Open.*

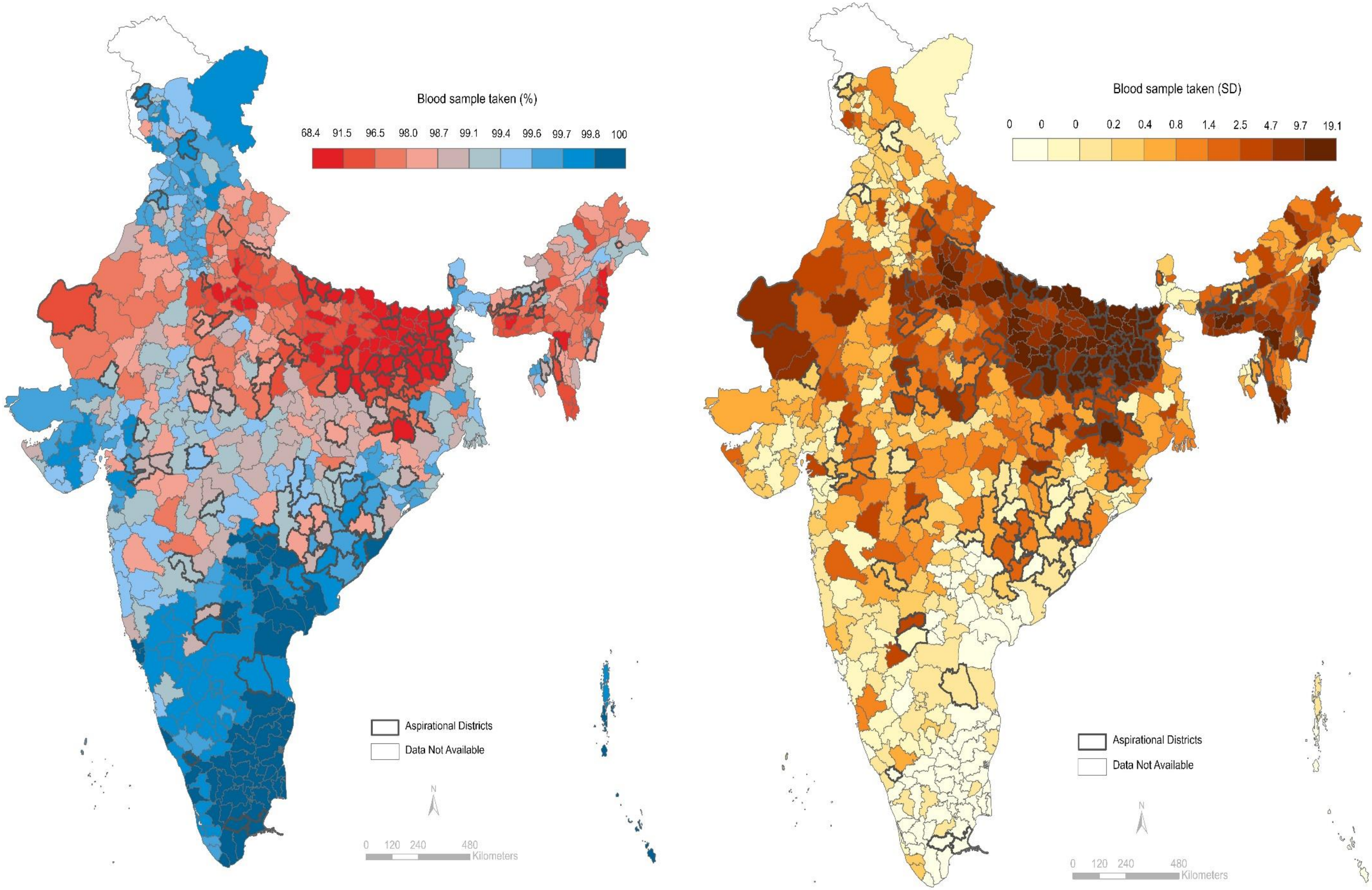

ANC: blood sample taken

© 2022 Lee H-Y et al. *JAMA Network Open.*

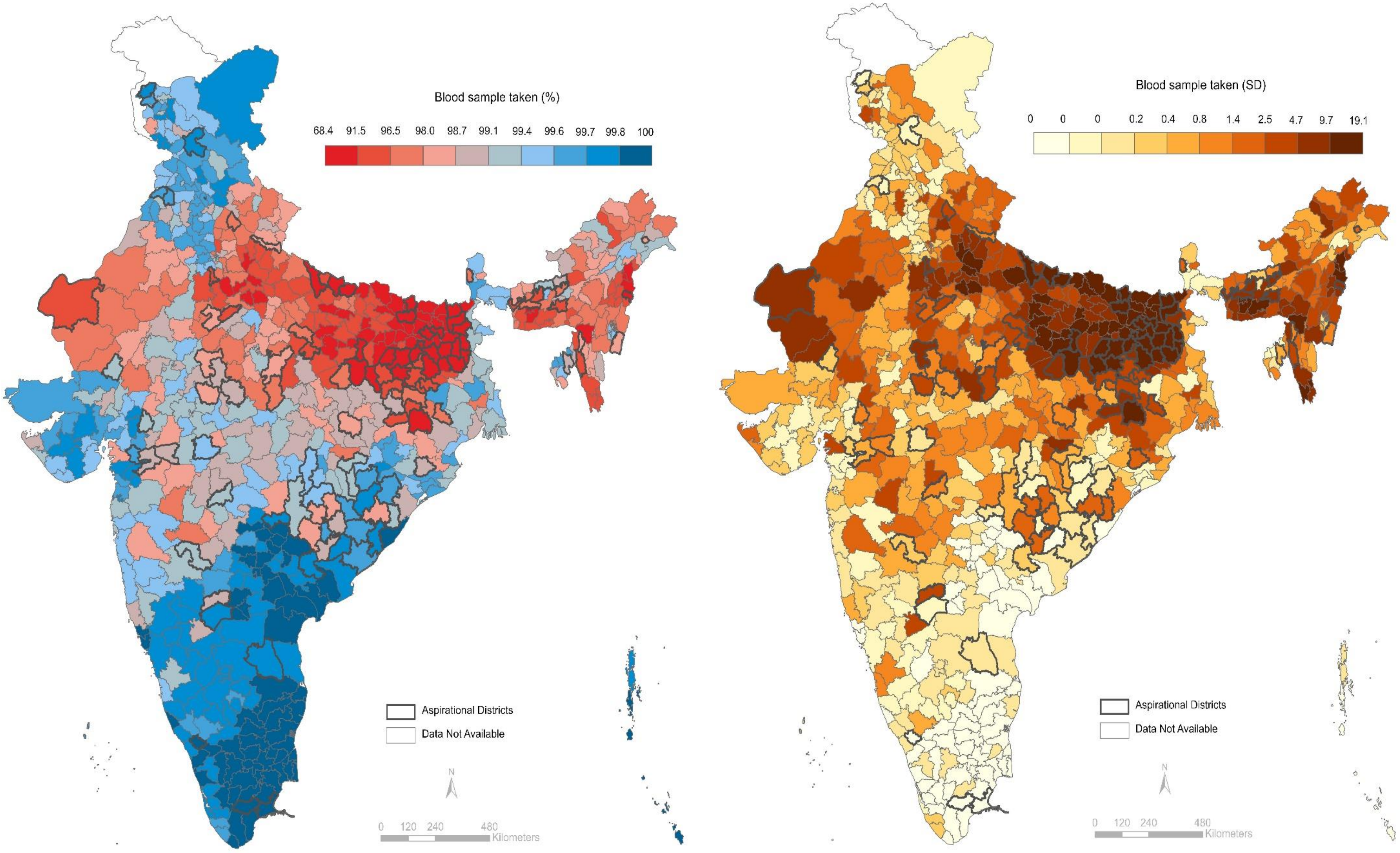

ANC: ultrasound test taken

© 2022 Lee H-Y et al. *JAMA Network Open.*

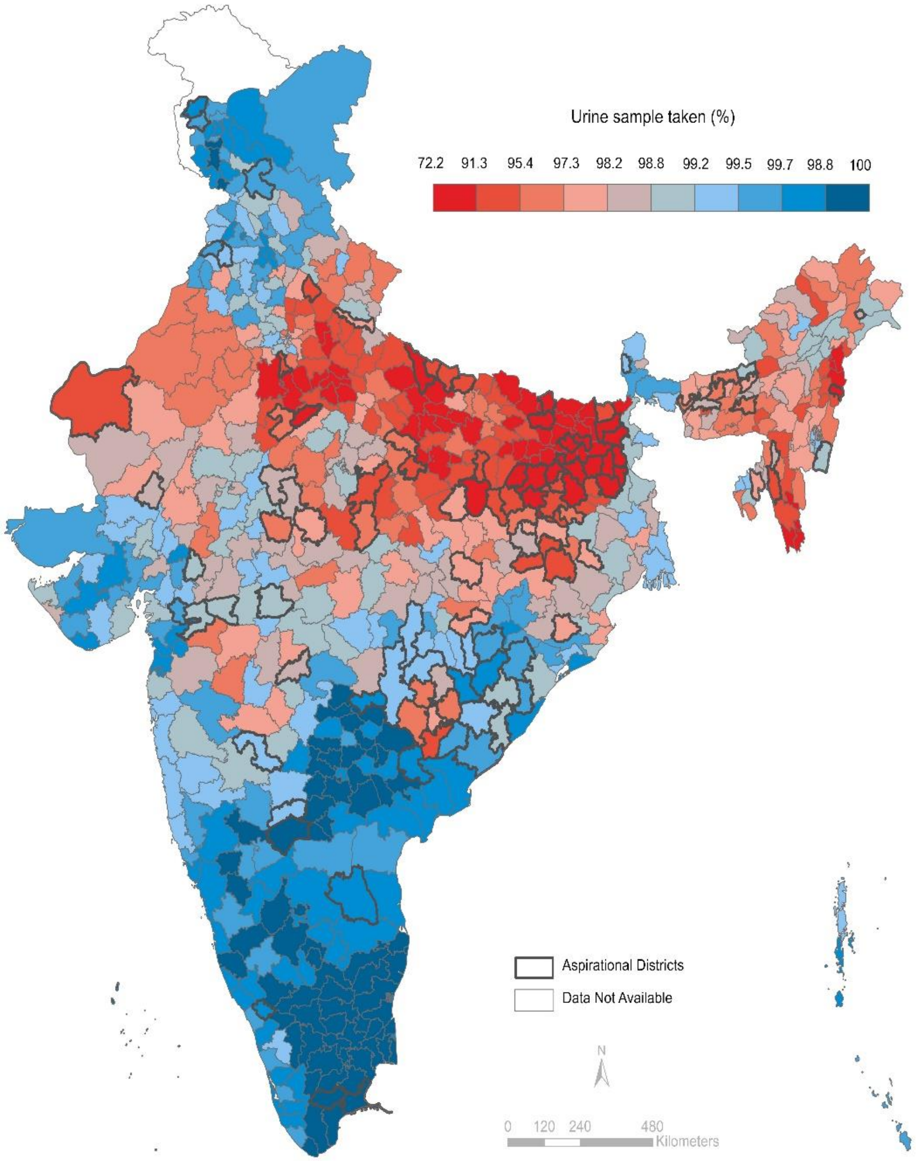

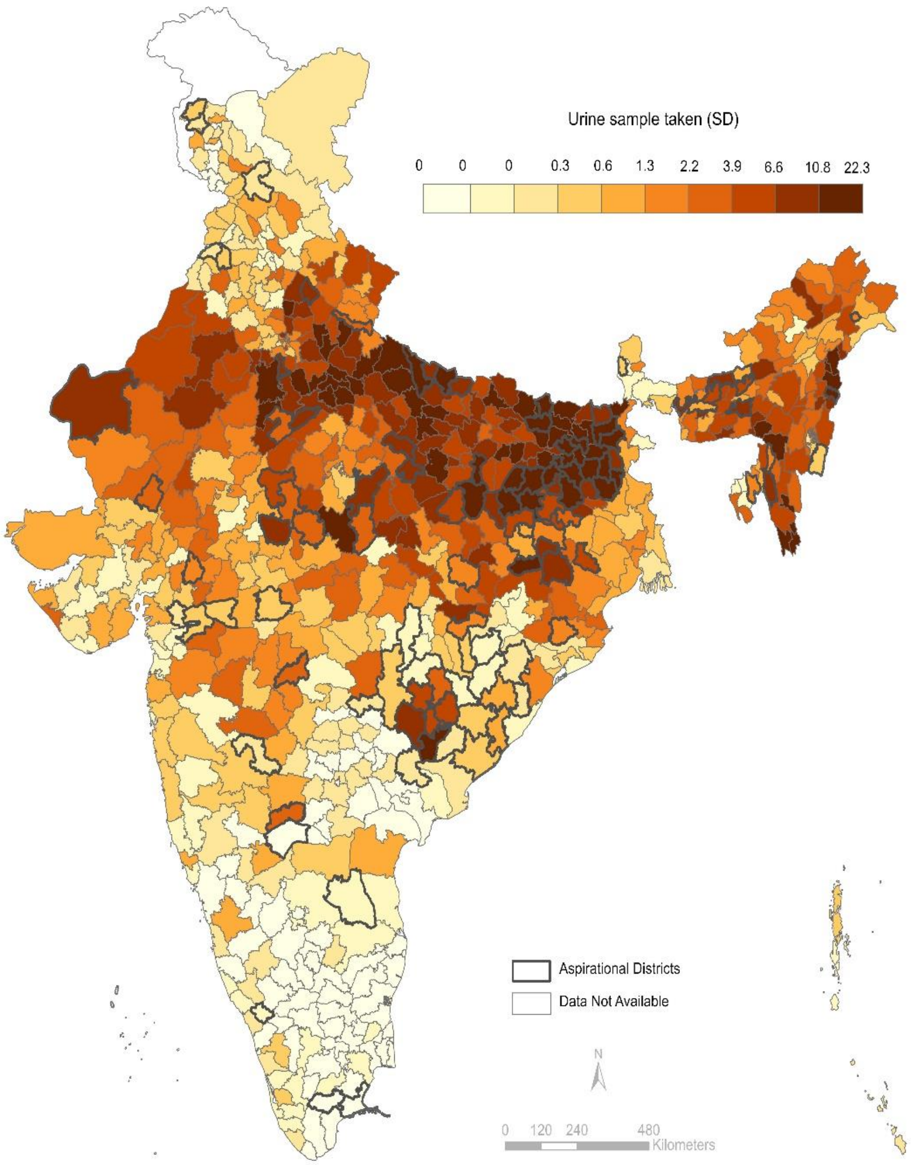

ANC: urine sample taken

© 2022 Lee H-Y et al. *JAMA Network Open.*

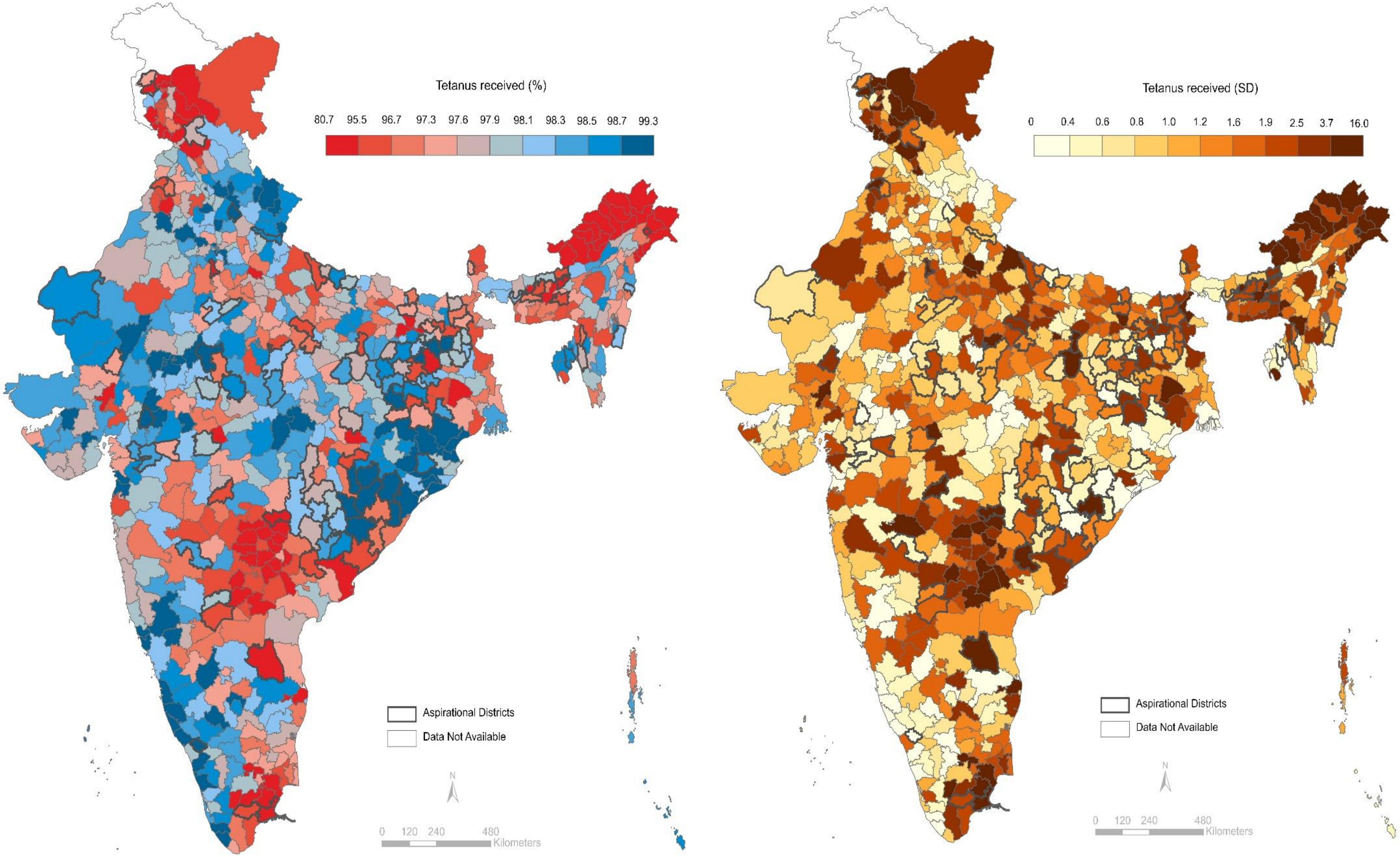

ANC: tetanus injection received

© 2022 Lee H-Y et al. *JAMA Network Open.*

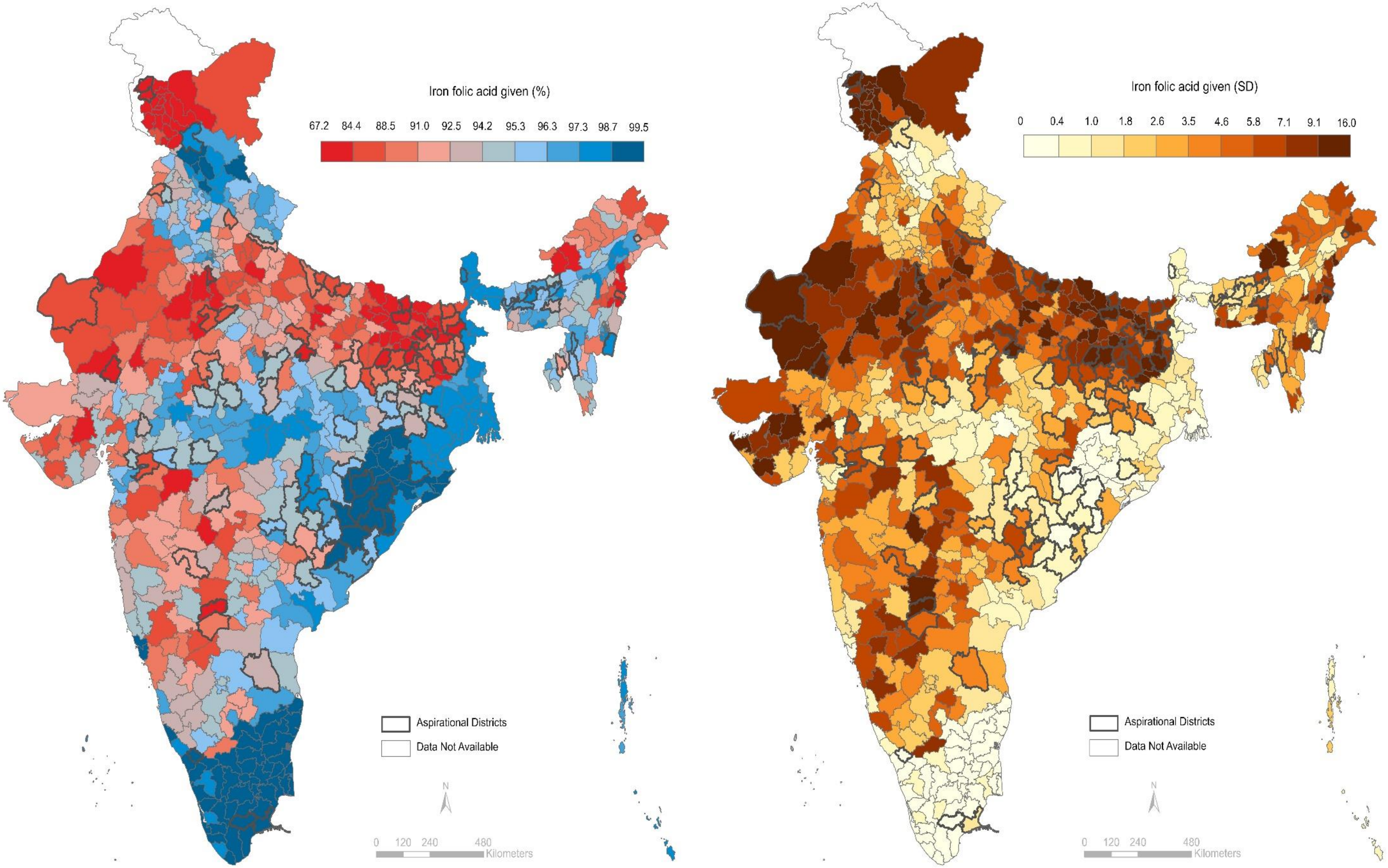

ANC: Iron given or bought

© 2022 Lee H-Y et al. *JAMA Network Open.*

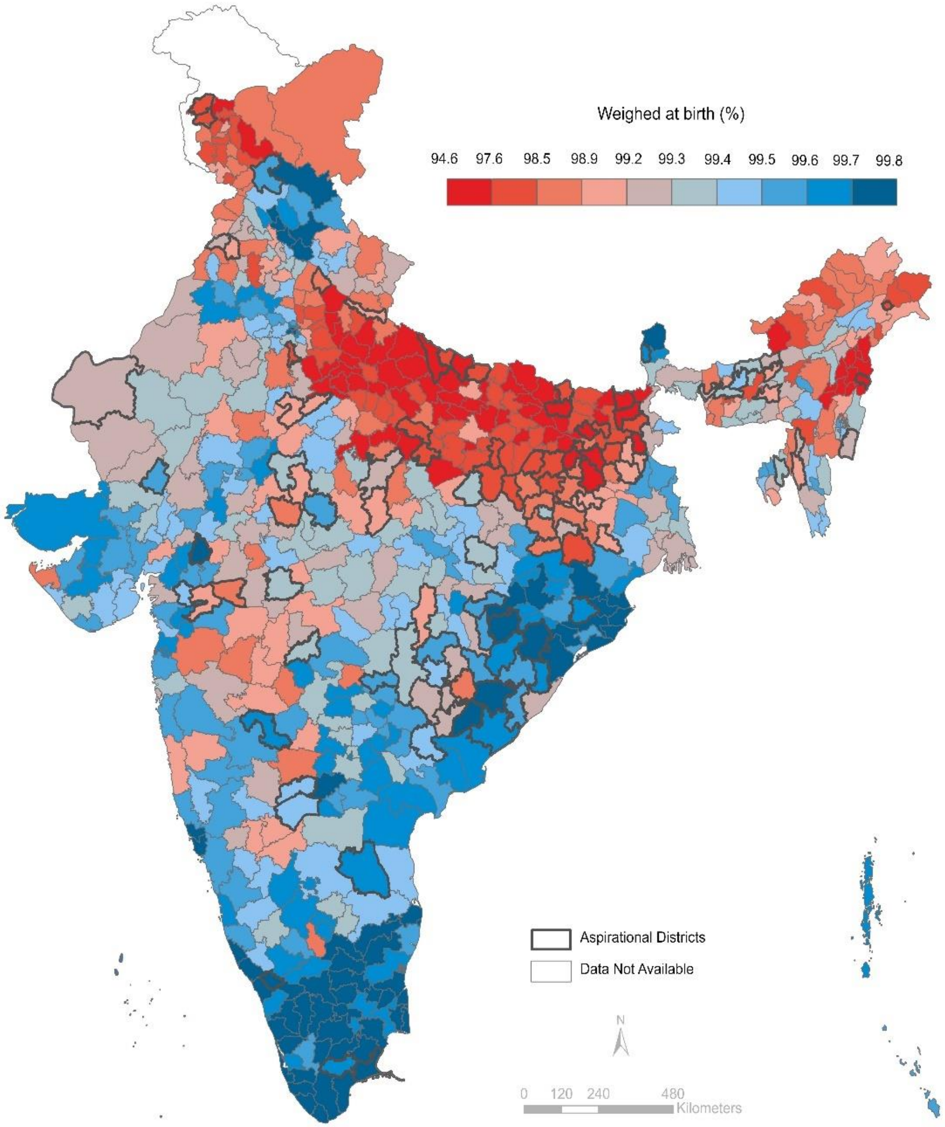

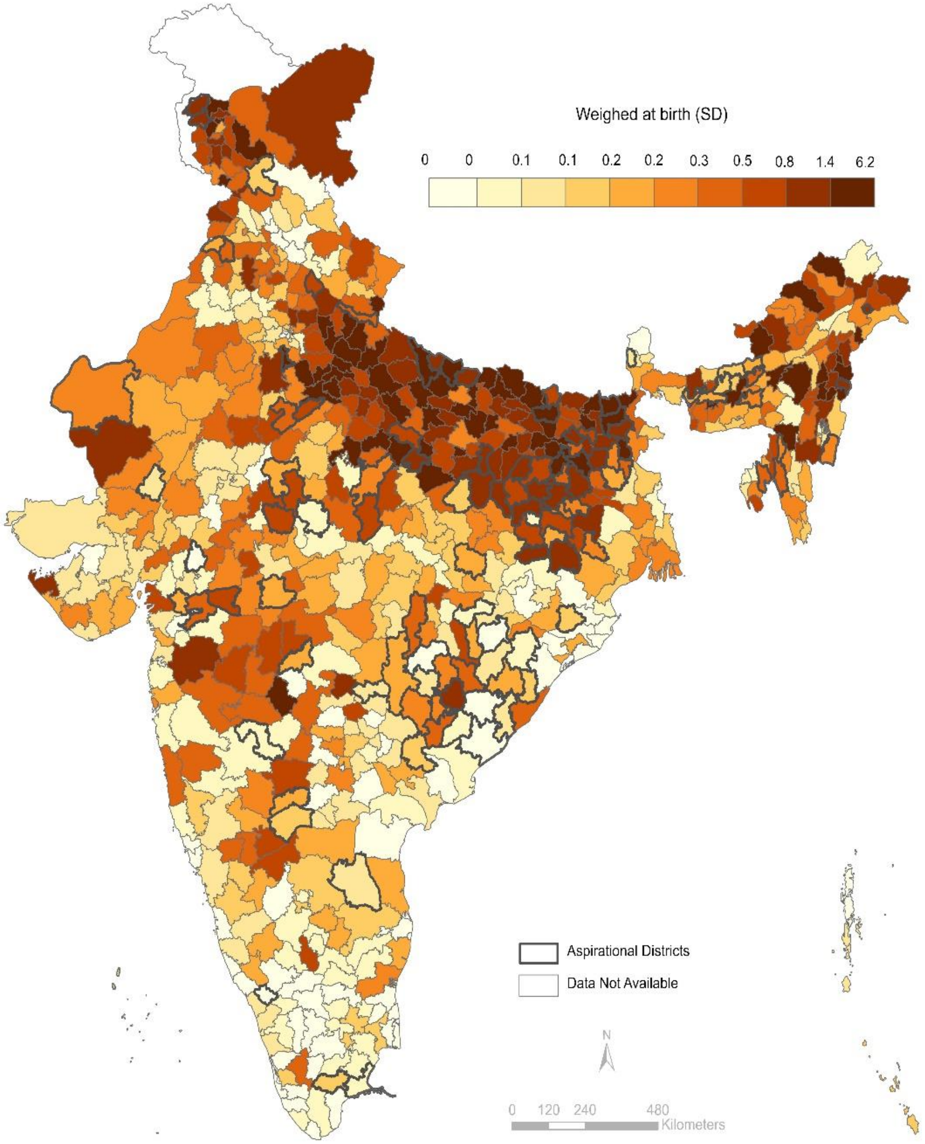

PNC: weighed at birth

© 2022 Lee H-Y et al. *JAMA Network Open.*

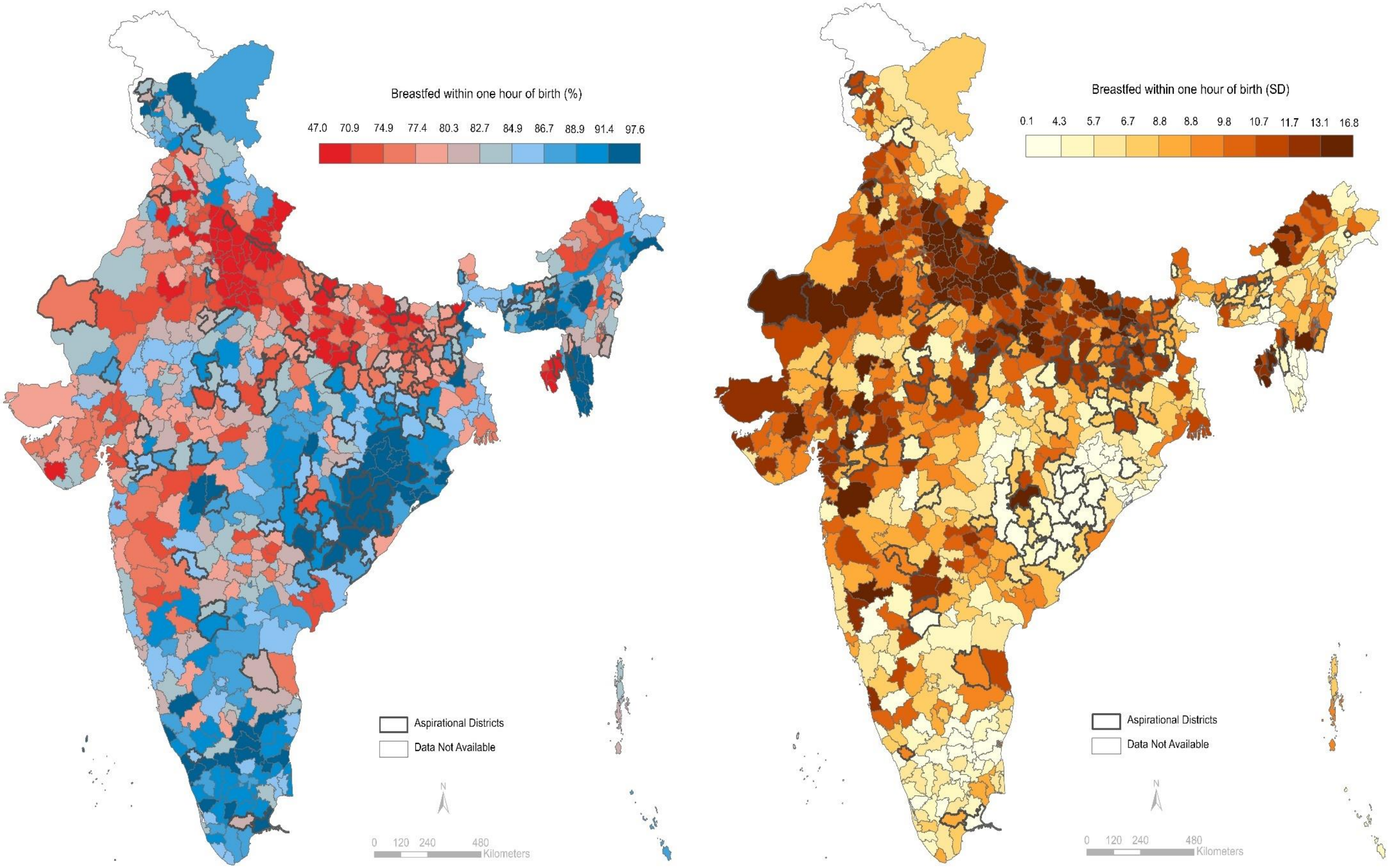

PNC: put to breast ≤ 1hour

© 2022 Lee H-Y et al. *JAMA Network Open.*

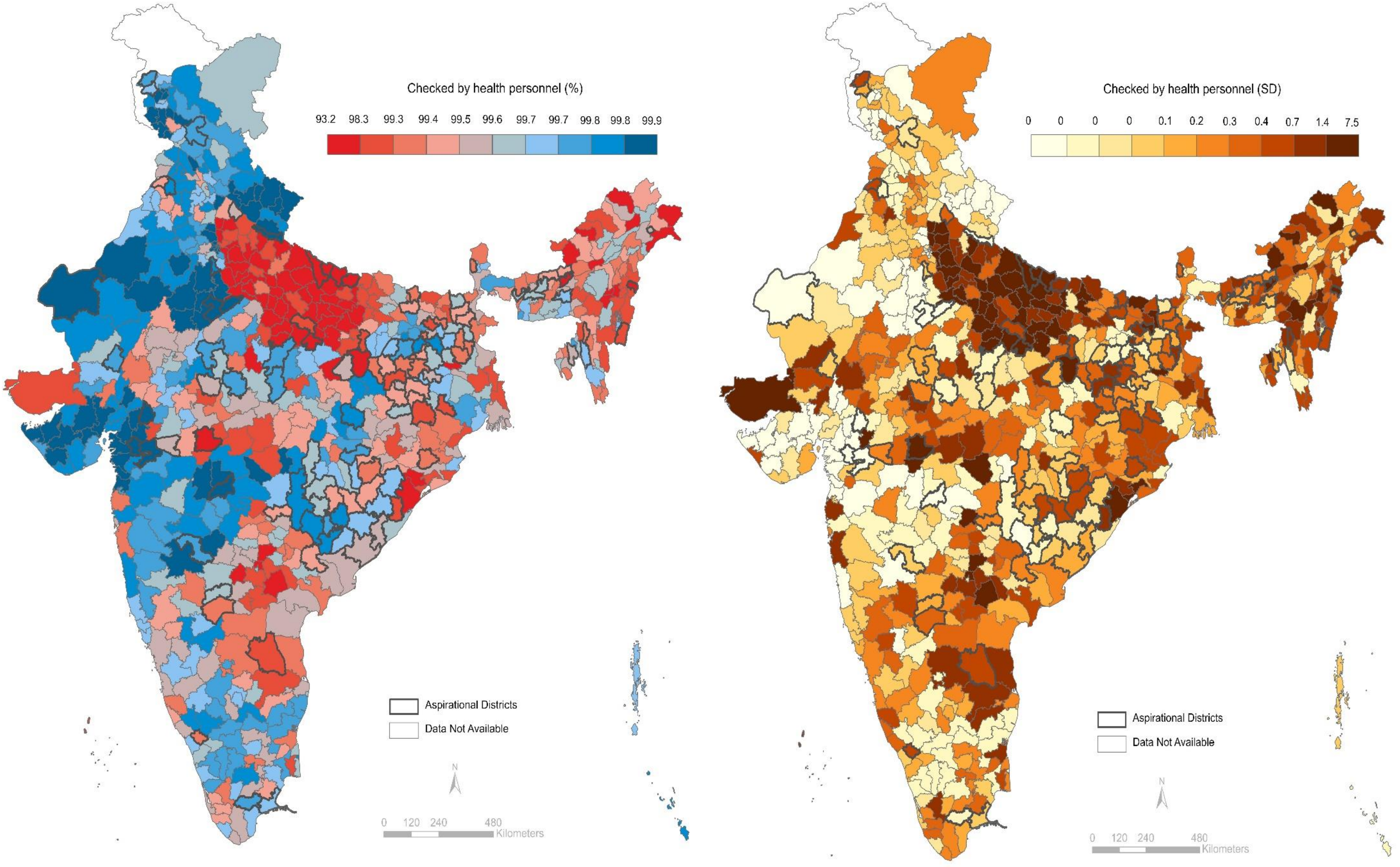

PNC: checked by health professionals

© 2022 Lee H-Y et al. *JAMA Network Open.*

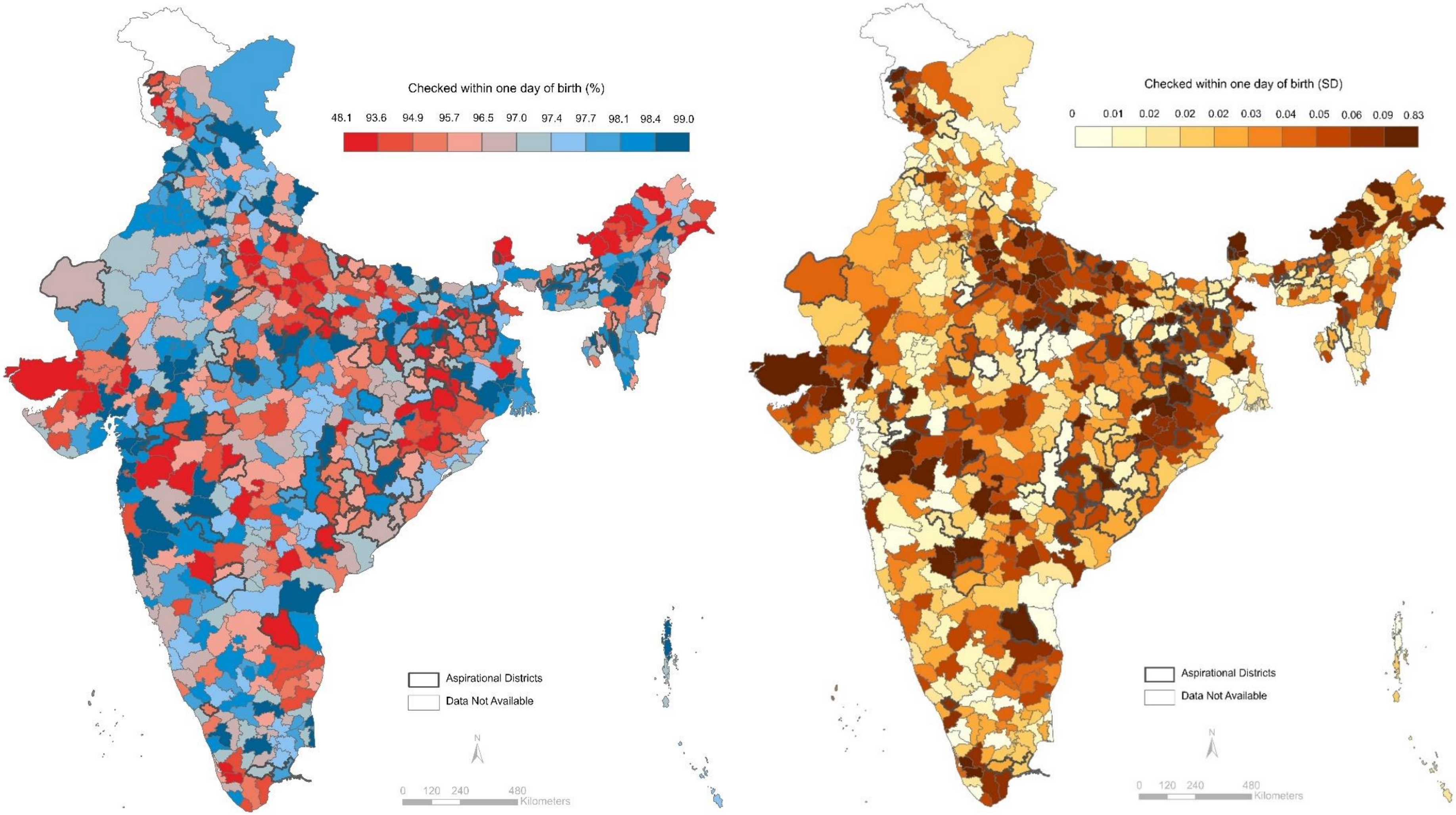

PNC: checked ≤ 24 hours

© 2022 Lee H-Y et al. *JAMA Network Open.*

eFigure 4. Correlation between prevalence of individual care contents received and within-district, between-clusters SD of them across 707 districts in India.

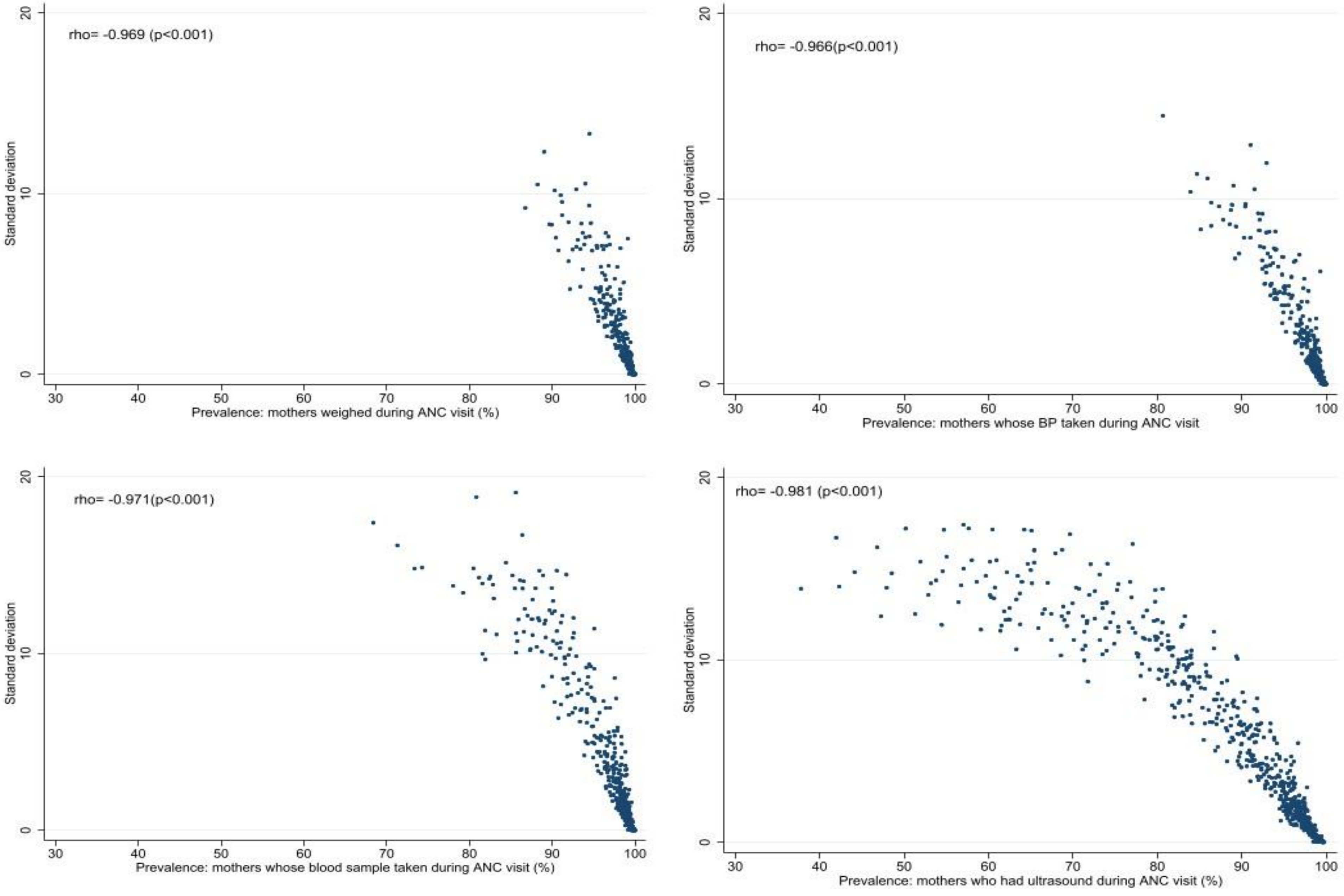

© 2022 Lee H-Y et al. *JAMA Network Open.*

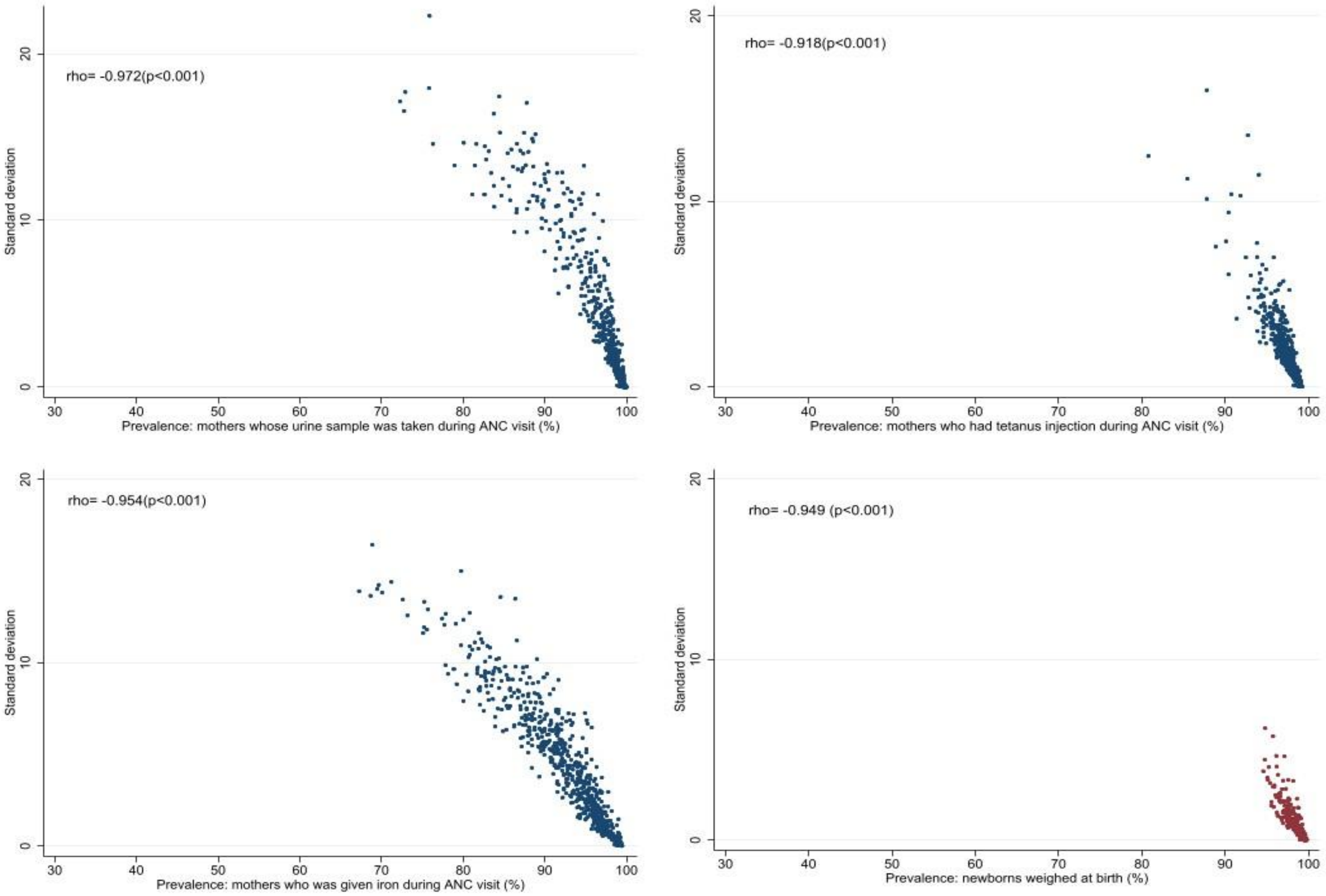

© 2022 Lee H-Y et al. *JAMA Network Open.*

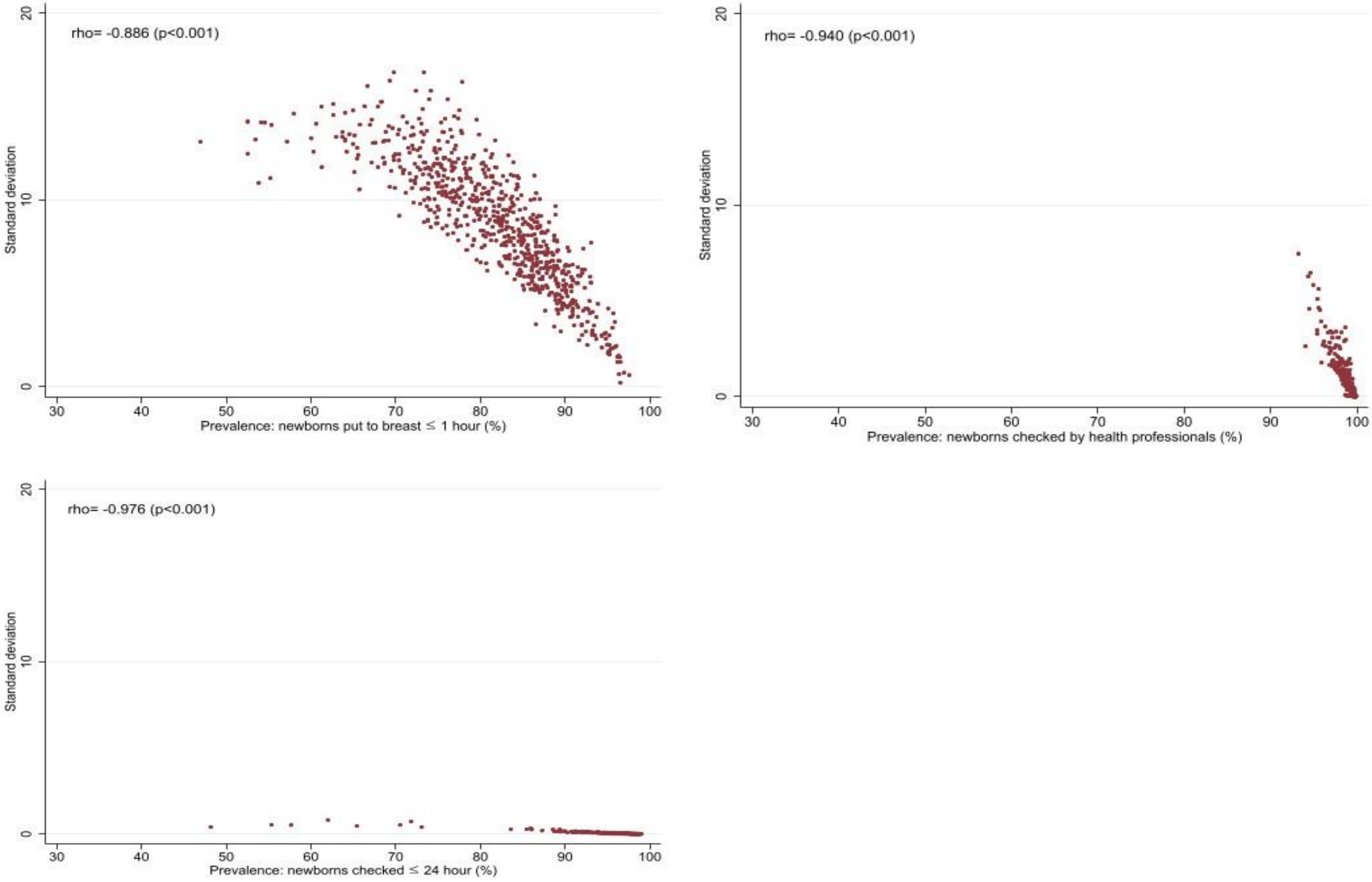

© 2022 Lee H-Y et al. *JAMA Network Open.*

eTable 1. Components of antenatal care (ANC) and postnatal care (PNC) to construct the composite score for maternal and newborn care quality

**Composite quality indicators (N=123,257, Mean = 93.5, SD=2.8)**					
	N	(%)		N	(%)
Weighed			Weighed at birth		
No	3,323	(2.5%)	No	2,793	(1.8%)
Yes	131,096	(97.5%)	Yes	152,141	(96.8%)
Blood pressure taken			Missing	2,203	(1.4%)
No	9,261	(3.2%)	Put to breast ≤ 1 hour		
Yes	130,156	(96.8%)	No	32,585	(20.7%)
Urine sample taken			Yes	115,348	(73.4%)
No	8,236	(6.1%)	Missing	9,204	(5.9%)
Yes	126,183	(93.9%)	Check by health professional		
Blood sample taken			No	2,112	(1.3%)
No	7,212	(5.4%)	Yes	136,190	(86.7%)
Yes	127,207	(94.6%)	Missing	18,835	(12.0%)
Tetanus injection			Postnatal check ≤ 24 hour		
No	5,494	(4.1%)	No	5,542	(3.5%)
Yes	128,369	(95.5%)	Yes	130,951	(83.3%)
Missing	556	(0.4%)	Missing	20,644	(13.1%)
Iron given or bought					
No	14,428	(10.7%)			
Yes	119,694	(89.0%)			
Missing	297	(0.2%)			
Ultrasound test taken					
No	20,092	(14.9%)			
Yes	114,327	(85.1%)			

Sample sizes for estimates of individual contents of ANC and PNC are different due to different inclusion criteria for ANC and PNC quality estimates

© 2022 Lee H-Y et al. *JAMA Network Open.*

eTable 2. District-level precision-weighted prevalence of individual contents of care received and composite quality score and standard deviation (SD) of them between clusters.

		Mean	Median	Min	Max
ANC	District-level prevalence				
	Weighed	98.9	99.6	86.8	100.0
	Blood pressure taken	98.5	99.4	80.7	100.0
	Blood sample taken	97.3	99.1	68.4	100.0
	Ultrasound test taken	89.2	95.1	37.8	99.7
	Urine sample taken	97.0	98.8	72.3	100.0
	Tetanus injection received	97.4	97.9	80.8	99.3
	Iron given or bought	92.6	94.2	67.2	99.5
	SD				
	Being weighed	1.14	2.13	0.00	13.33
	Blood pressure taken	1.34	2.37	0.00	14.49
	Blood sample taken	2.67	3.98	0.00	19.11
	Ultrasound test taken	5.02	4.88	0.00	17.42
	Urine sample taken	3.31	4.31	0.00	22.26
	Tetanus injection received	1.73	1.77	0.03	16.01
	Iron given or bought	4.23	3.36	0.02	16.44
PNC	District-level prevalence				
	Weighed at birth	99.0	99.3	94.6	99.8
	Put to breast ≤ 1hr	81.6	82.7	47.0	97.6
	Checked by health professional	99.3	99.5	97.7	99.8
	Checked ≦ 1hr	96.0	97.0	48.1	99.0
	SD				

© 2022 Lee H-Y et al. *JAMA Network Open.*

	Weighed at birth	0.52	0.78	0.00	6.19
	Put to breast ≤ 1hr	8.74	3.31	0.19	16.84
	Checked by health professional	0.52	0.91	0.09	7.48
	Checked ≤ 1day	0.05	0.07	0.00	0.83
Composite score	Average score	93.5	94.2	82.8	98.4
	SD	1.84	0.68	0.39	4.37

© 2022 Lee H-Y et al. *JAMA Network Open.*

eTable 3. District-level average composite score and within-district, between- clusters SD of them and their positions in the decile distribution, India, 2019-2020.

No	State	District	ADs	Composite score (%)		Decile		Districts requiring policy attention
				Score	SD	Score	SD	
1	Jammu & kashmir	Kupwara	Yes	94.1	2.01	5	7	No
2	Jammu & kashmir	Badgam	No	94.8	1.89	7	7	No
3	Ladakh	Leh(ladakh)	No	93.8	2.22	5	8	No
4	Ladakh	Kargil	No	94.8	1.7	7	5	No
5	Jammu & Kashmir	Punch	No	95.4	1.7	8	5	No
6	Jammu & Kashmir	Rajouri	No	92.5	2.03	3	7	No
7	Jammu & Kashmir	Kathua	No	94.8	1.42	7	4	No
8	Jammu & Kashmir	Baramula	Yes	91.4	3.54	3	10	Yes
9	Jammu & Kashmir	Bandipore	No	92.6	1.87	3	7	No
10	Jammu & Kashmir	Srinagar	No	94	1.8	5	6	No
11	Jammu & Kashmir	Ganderbal	No	94.6	1.7	6	5	No
12	Jammu & Kashmir	Pulwama	No	93.8	1.61	5	5	No
13	Jammu & Kashmir	Shupiyan	No	92.9	2	4	7	No
14	Jammu & Kashmir	Anantnag	No	93.1	2.14	4	8	No
15	Jammu & kashmir	Kulgam	No	94.6	1.39	6	3	No
16	Jammu & kashmir	Doda	No	93.5	1.96	4	7	No
17	Jammu & kashmir	Ramban	No	93.5	2.63	4	9	No
18	Jammu & kashmir	Kishtwar	No	92.5	1.59	3	5	No
19	Jammu & kashmir	Udhampur	No	94	2.05	5	8	No
20	Jammu & kashmir	Reasi	No	93.3	2.64	4	9	No
21	Jammu & kashmir	Jammu	No	95	1.69	7	5	No
22	Jammu & kashmir	Samba	No	94.5	1.3	6	3	No
23	Himachal	Chamba	Yes	96.5	1.18	9	2	No
24	Himachal	Kangra	No	95.1	1.39	7	3	No
25	Himachal	Lahul & spiti	No	96.3	1.27	9	3	No
26	Himachal	Kullu	No	96.6	1.02	10	1	No
27	Himachal	Mandi	No	95.2	1.66	7	5	No
28	Himachal	Hamirpur	No	95.5	1.26	8	3	No
29	Himachal	Una	No	91.9	1.57	3	4	No

© 2022 Lee H-Y et al. *JAMA Network Open.*

No	State	District	ADs	Composite score (%)		Decile		Districts requiring policy attention
				Score	SD	Score	SD	
30	Himachal	Bilaspur	No	95.7	1.08	8	1	No
31	Himachal	Solan	No	96	1.25	9	2	No
32	Himachal	Sirmaur	No	97.2	1.13	10	2	No
33	Himachal	Shimla	No	97.2	0.91	10	1	No
34	Himachal	Kinnaur	No	96.8	0.72	10	1	No
35	Punjab	Kapurthala	No	95.3	1.57	8	4	No
36	Punjab	Jalandhar	No	94.3	1.32	6	3	No
37	Punjab	Hoshiarpur	No	95.4	1.23	8	2	No
38	Punjab	Shahid bhagat singh nagar	No	95.4	1.53	8	4	No
39	Punjab	Fatehgarh sahib	No	94.9	1.38	7	3	No
40	Punjab	Ludhiana	No	93.5	1.76	4	6	No
41	Punjab	Moga	Yes	95.6	1.17	8	2	No
42	Punjab	Muktsar	No	95.9	0.98	9	1	No
43	Punjab	Faridkot	No	94	1.56	5	4	No
44	Punjab	Bathinda	No	93.7	1.93	5	7	No
45	Punjab	Mansa	No	96.3	0.85	9	1	No
46	Punjab	Patiala	No	94.5	1.79	6	6	No
47	Punjab	Amritsar	No	94.8	1.63	7	5	No
48	Punjab	Tarn taran	No	94.8	1.81	7	6	No
49	Punjab	Rupnagar	No	94.2	1.28	5	3	No
50	Punjab	Sahibzada ajit singh nagar	No	94.8	1.5	7	4	No
51	Punjab	Sangrur	No	95.4	1.35	8	3	No
52	Punjab	Barnala	No	95	1.65	7	5	No
53	Chandigarh	Chandigarhrh	No	96.7	1.16	10	2	No
54	Uttarakhand	Uttarkashi	No	95.6	1.53	8	4	No
55	Uttarakhand	Chamoli	No	94.4	1.58	6	5	No
56	Uttarakhand	Rudraprayag	No	95.9	1.16	9	2	No
57	Uttarakhand	Tehri garhwal	No	93.5	1.85	4	6	No
58	Uttarakhand	Dehradun	No	95.1	1.3	7	3	No
59	Uttarakhand	Garhwal	No	93.4	1.08	4	1	No

© 2022 Lee H-Y et al. *JAMA Network Open.*

No	State	District	ADs	Composite score (%)		Decile		Districts requiring policy attention
				Score	SD	Score	SD	
60	Uttarakhand	Pithoragarh	No	93.2	1.69	4	5	No
61	Uttarakhand	Bageshwar	No	93.8	1.35	5	3	No
62	Uttarakhand	Almora	No	93.3	1.67	4	5	No
63	Uttarakhand	Champawat	No	94.7	1.67	7	5	No
64	Uttarakhand	Nainital	No	92	1.78	3	6	No
65	Uttarakhand	Udham singh nagar	Yes	93.2	1.63	4	5	No
66	Uttarakhand	Hardwar	Yes	91.9	1.75	3	6	No
67	Haryana	Panchkula	No	96.8	1.04	10	1	No
68	Haryana	Ambala	No	93.9	1.78	5	6	No
69	Haryana	Yamunanagar	No	93.1	1.87	4	7	No
70	Haryana	Kurukshetra	No	95	1.76	7	6	No
71	Haryana	Kaithal	No	94.4	1.81	6	6	No
72	Haryana	Karnal	No	96.3	1.19	9	2	No
73	Haryana	Panipat	No	93.1	1.6	4	5	No
74	Haryana	Sonipat	No	95	1.34	7	3	No
75	Haryana	Jind	No	95.3	1.1	8	2	No
76	Haryana	Fatehabad	No	94.2	1.87	5	7	No
77	Haryana	Sirsa	No	95.4	1.19	8	2	No
78	Haryana	Hisar	No	95.5	1.83	8	6	No
79	Haryana	Rohtak	No	95.6	1.15	8	2	No
80	Haryana	Jhajjar	No	95.8	1.22	9	2	No
81	Haryana	Mahendragarh	No	93.8	1.84	5	6	No
82	Haryana	Rewari	No	94.1	1.83	5	6	No
83	Haryana	Gurgaon	No	94.8	1.54	7	4	No
84	Haryana	Mewat	Yes	91.3	2.96	2	10	Yes
85	Haryana	Faridabad	No	95.5	1.49	8	4	No
86	Haryana	Palwal	No	93.5	2.35	4	8	No
87	Rajasthan	Ganganagar	No	93.6	2.05	4	8	No
88	Rajasthan	Hanumangarh	No	94.2	2.2	6	8	No
89	Rajasthan	Bikaner	No	93.8	1.88	5	7	No

© 2022 Lee H-Y et al. *JAMA Network Open.*

No	State	District	ADs	Composite score (%)		Decile		Districts requiring policy attention
				Score	SD	Score	SD	
90	Rajasthan	Churu	No	92.5	2.62	3	9	Yes
91	Rajasthan	Jhunjhunun	No	93.5	1.86	4	7	No
92	Rajasthan	Alwar	No	90.1	2.67	2	9	Yes
93	Rajasthan	Bharatpur	No	89.7	2.84	2	10	Yes
94	Rajasthan	Dhaulpur	Yes	92	2.45	3	9	Yes
95	Rajasthan	Karauli	Yes	93	2.01	4	7	No
96	Rajasthan	Sawai madhopur	No	91.7	2.26	3	8	Yes
97	Rajasthan	Dausa	No	93.1	1.81	4	6	No
98	Rajasthan	Jaipur	No	92.7	1.77	4	6	No
99	Rajasthan	Sikar	No	92.7	2.67	3	9	Yes
100	Rajasthan	Nagaur	No	92.7	2.66	3	9	Yes
101	Rajasthan	Jodhpur	No	93.3	2.55	4	9	No
102	Rajasthan	Jaisalmer	Yes	92.4	2.84	3	10	Yes
103	Rajasthan	Barmer	No	94.2	2.86	6	10	No
104	Rajasthan	Jalor	No	93.4	2.71	4	9	No
105	Rajasthan	Sirohi	Yes	94.4	2.16	6	8	No
106	Rajasthan	Pali	No	94.6	1.95	6	7	No
107	Rajasthan	Ajmer	No	93.7	1.73	5	5	No
108	Rajasthan	Tonk	No	93.8	1.82	5	6	No
109	Rajasthan	Bundi	No	95.1	1.54	7	4	No
110	Rajasthan	Bhilwara	No	95.6	1.2	8	2	No
111	Rajasthan	Rajsamand	No	93.7	2.88	5	10	No
112	Rajasthan	Dungarpur	No	95.9	1.53	9	4	No
113	Rajasthan	Banswara	No	94.1	2.5	5	9	No
114	Rajasthan	Chittaurgarh	No	93.6	2.39	4	8	No
115	Rajasthan	Kota	No	95.8	1.32	8	3	No
116	Rajasthan	Baran	Yes	95.4	1.77	8	6	No
117	Rajasthan	Jhalawar	No	94.2	2.07	5	8	No
118	Rajasthan	Udaipur	No	94.9	1.76	7	6	No
119	Rajasthan	Pratapgarh	No	95.7	1.42	8	4	No
120	Uttar pradesh	Saharanpur	No	92.6	1.78	3	6	No

© 2022 Lee H-Y et al. *JAMA Network Open.*

No	State	District	ADs	Composite score (%)		Decile		Districts requiring policy attention
				Score	SD	Score	SD	
121	Uttar pradesh	Bijnor	No	89.2	1.75	1	6	No
122	Uttar pradesh	Rampur	No	90.7	2.82	2	9	Yes
123	Uttar pradesh	Jyotiba phule nagar	No	88.8	3.49	1	10	Yes
124	Uttar pradesh	Meerut	No	91.2	2.02	2	7	No
125	Uttar pradesh	Baghpat	No	90.6	2.27	2	8	Yes
126	Uttar pradesh	Gautam buddha nagar	No	92.4	1.67	3	5	No
127	Uttar pradesh	Bulandshahr	No	91	2.04	2	7	No
128	Uttar pradesh	Aligarh	No	90.9	2.3	2	8	Yes
129	Uttar pradesh	Mahamaya nagar	No	85.6	3.66	1	10	Yes
130	Uttar pradesh	Mathura	No	86.7	3.01	1	10	Yes
131	Uttar pradesh	Agra	No	88	2.83	1	9	Yes
132	Uttar pradesh	Firozabad	No	87.6	2.97	1	10	Yes
133	Uttar pradesh	Mainpuri	No	87.7	3.69	1	10	Yes
134	Uttar pradesh	Bareilly	No	90.7	2.78	2	9	Yes
135	Uttar pradesh	Pilibhit	No	91.9	2.1	3	8	Yes
136	Uttar pradesh	Shahjahanpur	No	90.3	2.75	2	9	Yes
137	Uttar pradesh	Kheri	No	88.2	3.39	1	10	Yes
138	Uttar pradesh	Sitapur	No	87.6	3.74	1	10	Yes
139	Uttar pradesh	Hardoi	No	87.9	3.24	1	10	Yes
140	Uttar pradesh	Unnao	No	85.1	3.05	1	10	Yes
141	Uttar pradesh	Lucknow	No	90.7	2.42	2	9	Yes
142	Uttar pradesh	Farrukhabad	No	91.1	2.79	2	9	Yes
143	Uttar pradesh	Kannauj	No	90.3	3.38	2	10	Yes
144	Uttar pradesh	Etawah	No	91.7	2.11	3	8	Yes
145	Uttar pradesh	Auraiya	No	90.7	2.85	2	10	Yes
146	Uttar pradesh	Kanpur dehat	No	92.4	2.22	3	8	Yes
147	Uttar pradesh	Kanpur nagar	No	92.3	1.71	3	5	No
148	Uttar pradesh	Jalaun	No	92.2	2.02	3	7	No
149	Uttar pradesh	Jhansi	No	90.8	2.71	2	9	Yes
150	Uttar pradesh	Lalitpur	No	91.5	2.07	3	8	Yes
151	Uttar pradesh	Hamirpur	No	91.2	3.22	2	10	Yes

© 2022 Lee H-Y et al. *JAMA Network Open.*

No	State	District	ADs	Composite score (%)		Decile		Districts requiring policy attention
				Score	SD	Score	SD	
152	Uttar pradesh	Mahoba	No	90.7	3.38	2	10	Yes
153	Uttar pradesh	Banda	No	89.1	3.66	1	10	Yes
154	Uttar pradesh	Chitrakoot	Yes	88.8	2.4	1	9	Yes
155	Uttar pradesh	Fatehpur	Yes	88.5	3.05	1	10	Yes
156	Uttar pradesh	Pratapgarh	No	89	2.18	1	8	Yes
157	Uttar pradesh	Kaushambi	No	89.4	2.73	2	9	Yes
158	Uttar pradesh	Allahabad	No	87.4	2.82	1	9	Yes
159	Uttar pradesh	Bara banki	No	87.2	3.82	1	10	Yes
160	Uttar pradesh	Faizabad	No	88.4	2.54	1	9	Yes
161	Uttar pradesh	Ambedkar nagar	No	88.6	2.94	1	10	Yes
162	Uttar pradesh	Bahraich	Yes	86.2	3.34	1	10	Yes
163	Uttar pradesh	Shrawasti	Yes	89.8	3.02	2	10	Yes
164	Uttar pradesh	Balrampur	Yes	87.5	3.38	1	10	Yes
165	Uttar pradesh	Gonda	No	89.3	2.64	1	9	Yes
166	Uttar pradesh	Siddharthnagar	Yes	89.4	2.85	2	10	Yes
167	Uttar pradesh	Basti	No	92.8	3.22	4	10	No
168	Uttar pradesh	Sant kabir nagar	No	92.1	2.35	3	8	Yes
169	Uttar pradesh	Mahrajganj	No	89.6	2.88	2	10	Yes
170	Uttar pradesh	Gorakhpur	No	91.4	2.61	3	9	Yes
171	Uttar pradesh	Kushinagar	No	89.4	2.96	2	10	Yes
172	Uttar pradesh	Deoria	No	89.7	3.3	2	10	Yes
173	Uttar pradesh	Azamgarh	No	88.9	2.96	1	10	Yes
174	Uttar pradesh	Mau	No	89.8	2.63	2	9	Yes
175	Uttar pradesh	Ballia	No	92.1	1.65	3	5	No
176	Uttar pradesh	Jaunpur	No	91.4	2.67	3	9	Yes
177	Uttar pradesh	Ghazipur	No	90.1	3.37	2	10	Yes
178	Uttar pradesh	Chandauli	Yes	87.7	3.74	1	10	Yes
179	Uttar pradesh	Varanasi	No	90	2.21	2	8	Yes
180	Uttar pradesh	Sant ravidas nagar (bhadohi)	No	86.7	3.27	1	10	Yes
181	Uttar pradesh	Mirzapur	No	89.1	2.58	1	9	Yes

© 2022 Lee H-Y et al. *JAMA Network Open.*

No	State	District	ADs	Composite score (%)		Decile		Districts requiring policy attention
				Score	SD	Score	SD	
182	Uttar pradesh	Sonbhadra	Yes	87.3	3.42	1	10	Yes
183	Uttar pradesh	Etah	No	85.6	3.59	1	10	Yes
184	Uttar pradesh	Kanshiram nagar	No	85.6	3.69	1	10	Yes
185	Bihar	Pashchim champaran	No	84.1	3.04	1	10	Yes
186	Bihar	Purba champaran	No	86.2	3.11	1	10	Yes
187	Bihar	Sheohar	No	88.7	2.9	1	10	Yes
188	Bihar	Sitamarhi	Yes	87.5	2.6	1	9	Yes
189	Bihar	Madhubani	No	88.1	2.37	1	8	Yes
190	Bihar	Supaul	No	86	2.95	1	10	Yes
191	Bihar	Araria	Yes	88.4	3.41	1	10	Yes
192	Bihar	Kishanganj	No	82.8	3.27	1	10	Yes
193	Bihar	Purnia	Yes	85.5	2.57	1	9	Yes
194	Bihar	Katihar	Yes	85.6	3.09	1	10	Yes
195	Bihar	Madhepura	No	84.9	2.76	1	9	Yes
196	Bihar	Saharsa	No	84.1	2.41	1	9	Yes
197	Bihar	Darbhanga	No	84.2	4.05	1	10	Yes
198	Bihar	Muzaffarpur	Yes	86.4	3.3	1	10	Yes
199	Bihar	Gopalganj	No	87.7	3.19	1	10	Yes
200	Bihar	Siwan	No	88.1	2.63	1	9	Yes
201	Bihar	Saran	No	89.2	1.98	1	7	No
202	Bihar	Vaishali	No	86.6	2.73	1	9	Yes
203	Bihar	Samastipur	No	84.9	3.24	1	10	Yes
204	Bihar	Begusarai	Yes	86.8	2.48	1	9	Yes
205	Bihar	Khagaria	Yes	84.8	3.48	1	10	Yes
206	Bihar	Bhagalpur	No	87.2	2.81	1	9	Yes
207	Bihar	Banka	Yes	88.3	2.84	1	10	Yes
208	Bihar	Munger	No	88.3	2.69	1	9	Yes
209	Bihar	Lakhisarai	No	85.9	2.8	1	9	Yes
210	Bihar	Sheikhpura	Yes	86	3.49	1	10	Yes
211	Bihar	Nalanda	No	87.7	2.68	1	9	Yes

© 2022 Lee H-Y et al. *JAMA Network Open.*

No	State	District	ADs	Composite score (%)		Decile		Districts requiring policy attention
				Score	SD	Score	SD	
212	Bihar	Patna	No	86.3	2.91	1	10	Yes
213	Bihar	Bhojpur	No	87.6	3.31	1	10	Yes
214	Bihar	Buxar	No	88.2	2.75	1	9	Yes
215	Bihar	Kaimur (bhabua)	No	91.3	1.79	2	6	No
216	Bihar	Rohtas	No	89.7	2.59	2	9	Yes
217	Bihar	Aurangabad	Yes	89.3	2.65	1	9	Yes
218	Bihar	Gaya	Yes	88.9	2.74	1	9	Yes
219	Bihar	Nawada	Yes	86.9	2.72	1	9	Yes
220	Bihar	Jamui	Yes	87.1	2.96	1	10	Yes
221	Bihar	Jehanabad	No	88.7	2.08	1	8	Yes
222	Bihar	Arwal	No	89.5	2.65	2	9	Yes
223	Sikkim	North district	No	94.3	1.98	6	7	No
224	Sikkim	West district	Yes	94.9	1.57	7	4	No
225	Sikkim	South district	No	95.5	1.58	8	5	No
226	Sikkim	East district	No	94.2	1.25	6	2	No
227	Arunachal pradesh	Tawang	No	93.1	2.34	4	8	No
228	Arunachal pradesh	West kameng	No	91.3	2.85	3	10	Yes
229	Arunachal pradesh	East kameng	No	90	2.12	2	8	Yes
230	Arunachal pradesh	Papum pare	No	92.3	2.1	3	8	Yes
231	Arunachal pradesh	Upper subansiri	No	90.8	3.12	2	10	Yes
232	Arunachal pradesh	Upper siang	No	90.9	2.92	2	10	Yes
233	Arunachal pradesh	Changlang	No	94.9	1.52	7	4	No
234	Arunachal pradesh	Lower subansiri	No	92.7	1.75	3	6	No
235	Arunachal pradesh	Dibang valley	No	92.7	1.79	4	6	No
236	Arunachal pradesh	Lower dibang valley	No	93.9	1.68	5	5	No
237	Arunachal pradesh	Anjaw	No	92.2	2.26	3	8	Yes
238	Nagaland	Mon	No	90.4	1.73	2	6	No
239	Nagaland	Mokokchung	No	92.1	1.71	3	5	No
240	Nagaland	Zunheboto	No	91.4	2	3	7	No
241	Nagaland	Wokha	No	91.1	2.08	2	8	Yes
242	Nagaland	Dimapur	No	92.2	1.83	3	6	No

© 2022 Lee H-Y et al. *JAMA Network Open.*

No	State	District	ADs	Composite score (%)		Decile		Districts requiring policy attention
				Score	SD	Score	SD	
243	Nagaland	Phek	No	91	1.76	2	6	No
244	Nagaland	Tuensang	No	91.8	2.23	3	8	Yes
245	Nagaland	Longleng	No	90.8	1.68	2	5	No
246	Nagaland	Kiphire	Yes	90.6	2.23	2	8	Yes
247	Nagaland	Kohima	No	93.5	1.71	4	5	No
248	Nagaland	Peren	No	92.6	1.59	3	5	No
249	Manipur	Senapati	No	95.4	1.64	8	5	No
250	Manipur	Tamenglong	No	94.3	1.64	6	5	No
251	Manipur	Churachandpur	No	94	2.18	5	8	No
252	Manipur	Bishnupur	No	94.3	1.83	6	6	No
253	Manipur	Thoubal	No	95.7	1.32	8	3	No
254	Manipur	Imphal west	No	95.9	1.49	9	4	No
255	Manipur	Imphal east	No	95.1	1.64	7	5	No
256	Manipur	Ukhrul	No	94.9	1.48	7	4	No
257	Manipur	Chandel	Yes	95.8	1.62	8	5	No
258	Mizoram	Mamit	Yes	91.6	2.17	3	8	Yes
259	Mizoram	Kolasib	No	92.9	2.23	4	8	No
260	Mizoram	Aizawl	No	89.4	2.36	2	8	Yes
261	Mizoram	Champhai	No	93	1.96	4	7	No
262	Mizoram	Serchhip	No	90.7	1.84	2	6	No
263	Mizoram	Lunglei	No	91	2.59	2	9	Yes
264	Mizoram	Lawngtlai	No	91	2.13	2	8	Yes
265	Mizoram	Saiha	No	89.2	1.93	1	7	No
266	Tripura	Dhalai	Yes	90.4	2.11	2	8	Yes
267	Meghalay	South garo hills	No	92.3	2.21	3	8	Yes
268	Meghalay	Ribhoi	Yes	93.2	2.02	4	7	No
269	Meghalay	East khasi hills	No	93.7	1.82	5	6	No
270	Assam	Kokrajhar	No	94.6	2.4	6	9	No
271	Assam	Goalpara	Yes	94.6	1.67	6	5	No
272	Assam	Barpeta	Yes	94.6	2.19	6	8	No
273	Assam	Morigaon	No	96.1	1.35	9	3	No

© 2022 Lee H-Y et al. *JAMA Network Open.*

No	State	District	ADs	Composite score (%)		Decile		Districts requiring policy attention
				Score	SD	Score	SD	
274	Assam	Lakhimpur	No	95.3	1.97	8	7	No
275	Assam	Dhemaji	No	95.8	1.15	8	2	No
276	Assam	Tinsukia	No	96.1	1.45	9	4	No
277	Assam	Dibrugarh	No	96.5	1.1	9	2	No
278	Assam	Golaghat	No	95.4	1.32	8	3	No
279	Assam	Dima hasao	No	93.9	2.69	5	9	No
280	Assam	Cachar	No	90.8	3.92	2	10	Yes
281	Assam	Karimganj	No	93	2.39	4	9	No
282	Assam	Hailakandi	Yes	93.6	2.19	4	8	No
283	Assam	Bongaigaon	No	93.4	2	4	7	No
284	Assam	Chirang	No	95.5	1.69	8	5	No
285	Assam	Kamrup	No	92.7	3.36	3	10	Yes
286	Assam	Kamrup metropolitan	No	94.8	1.57	7	4	No
287	Assam	Nalbari	No	95.1	1.65	7	5	No
288	Assam	Baksa	Yes	93.2	2.41	4	9	No
289	Assam	Darrang	Yes	94	1.98	5	7	No
290	Assam	Udalguri	Yes	94	1.66	5	5	No
291	West bengal	Darjiling	No	96.4	1.15	9	2	No
292	West bengal	Jalpaiguri	No	97	1.26	10	3	No
293	West bengal	Koch bihar	No	95.9	1.31	9	3	No
294	West bengal	Uttar dinajpur	No	96.1	1.82	9	6	No
295	West bengal	Dakshin dinajpur	No	97.3	0.73	10	1	No
296	West bengal	Maldah	No	95.8	1.43	9	4	No
297	West bengal	Murshidabad	No	95.1	1.48	7	4	No
298	West bengal	Birbhum	No	95.6	1.39	8	3	No
299	West bengal	Nadia	No	95.9	0.86	9	1	No
300	West bengal	North twenty four parganas	No	95.8	1.3	8	3	No
301	West bengal	Hugli	No	94.3	1.72	6	5	No
302	West bengal	Bankura	No	93.7	2.02	5	7	No
303	West bengal	Puruliya	No	94.3	1.46	6	4	No

© 2022 Lee H-Y et al. *JAMA Network Open.*

No	State	District	ADs	Composite score (%)		Decile		Districts requiring policy attention
				Score	SD	Score	SD	
304	West bengal	Haora	No	95.8	1.34	9	3	No
305	West bengal	Kolkata	No	95.6	1.55	8	4	No
306	West bengal	South twenty four parganas	No	95.2	1.95	7	7	No
307	West bengal	Paschim medinipur	No	96.7	0.67	10	1	No
308	West bengal	Purba medinipur	No	92.9	1.7	4	5	No
309	Jharkhand	Garhwa	Yes	89.8	2.3	2	8	Yes
310	Jharkhand	Chatra	Yes	89.7	3.08	2	10	Yes
311	Jharkhand	Kodarma	No	91.7	3.02	3	10	Yes
312	Jharkhand	Giridih	Yes	90.1	2.64	2	9	Yes
313	Jharkhand	Deoghar	No	89.4	2.47	2	9	Yes
314	Jharkhand	Godda	Yes	90.7	3.04	2	10	Yes
315	Jharkhand	Sahibganj	Yes	89.6	2.38	2	8	Yes
316	Jharkhand	Pakur	Yes	90.8	2.6	2	9	Yes
317	Jharkhand	Dhanbad	No	91.5	2.73	3	9	Yes
318	Jharkhand	Bokaro	Yes	92.9	1.88	4	7	No
319	Jharkhand	Lohardaga	Yes	95	1.38	7	3	No
320	Jharkhand	Purbi singhbhum	Yes	94.4	1.5	6	4	No
321	Jharkhand	Palamu	Yes	91.3	2.35	2	8	Yes
322	Jharkhand	Latehar	Yes	92.3	2.39	3	9	Yes
323	Jharkhand	Hazaribagh	Yes	91.8	1.94	3	7	No
324	Jharkhand	Ramgarh	Yes	91.9	2.16	3	8	Yes
325	Jharkhand	Dumka	Yes	90.9	2.6	2	9	Yes
326	Jharkhand	Jamtara	No	90.1	2.26	2	8	Yes
327	Jharkhand	Ranchi	Yes	94.7	1.46	6	4	No
328	Jharkhand	Khunti	Yes	92.7	2.02	4	7	No
329	Jharkhand	Gumla	Yes	94.1	1.81	5	6	No
330	Jharkhand	Simdega	Yes	91.6	2.61	3	9	Yes
331	Jharkhand	Pashchimi singhbhum	Yes	91.5	2.79	3	9	Yes
332	Jharkhand	Saraikela-kharsawan	No	93.7	1.87	4	7	No
333	Odisha	Bargarh	No	97.2	0.94	10	1	No
334	Odisha	Jharsuguda	No	97.6	0.98	10	1	No

© 2022 Lee H-Y et al. *JAMA Network Open.*

No	State	District	ADs	Composite score (%)		Decile		Districts requiring policy attention
				Score	SD	Score	SD	
335	Odisha	Sambalpur	No	97.1	0.98	10	1	No
336	Odisha	Debagarh	No	96.7	1.2	10	2	No
337	Odisha	Sundargarh	No	95.2	1.76	7	6	No
338	Odisha	Kendujhar	No	95.4	1.72	8	5	No
339	Odisha	Mayurbhanj	No	95.5	1.48	8	4	No
340	Odisha	Baleshwar	No	95.8	1.72	9	5	No
341	Odisha	Bhadrak	No	96.7	1.25	10	2	No
342	Odisha	Kendrapara	No	97.2	1.24	10	2	No
343	Odisha	Jagatsinghapur	No	96.7	1.06	10	1	No
344	Odisha	Cuttack	No	97	1.04	10	1	No
345	Odisha	Jajapur	No	96.2	1.31	9	3	No
346	Odisha	Dhenkanal	Yes	96.5	1.09	9	1	No
347	Odisha	Anugul	No	96.8	1.15	10	2	No
348	Odisha	Nayagarh	No	98.3	0.65	10	1	No
349	Odisha	Khordha	No	96.9	1.06	10	1	No
350	Odisha	Puri	No	97.7	0.77	10	1	No
351	Odisha	Ganjam	No	95.9	1.76	9	6	No
352	Odisha	Gajapati	Yes	96.8	1.35	10	3	No
353	Odisha	Kandhamal	Yes	96.8	1.45	10	4	No
354	Odisha	Baudh	No	96.7	1.54	10	4	No
355	Odisha	Subarnapur	No	97.1	1.46	10	4	No
356	Odisha	Balangir	Yes	97.6	1.03	10	1	No
357	Odisha	Nuapada	Yes	96.8	1.06	10	1	No
358	Odisha	Kalahandi	Yes	97.4	1.23	10	2	No
359	Odisha	Rayagada	Yes	95.1	1.65	7	5	No
360	Odisha	Nabarangapur	Yes	96.4	1.6	9	5	No
361	Odisha	Koraput	Yes	96.8	0.9	10	1	No
362	Odisha	Malkangiri	Yes	96.4	1.32	9	3	No
363	Chhattisgarh	Koriya	No	94.9	1.73	7	6	No
364	Chhattisgarh	Jashpur	No	94.3	1.29	6	3	No
365	Chhattisgarh	Raigarh	No	94.8	1.59	7	5	No

© 2022 Lee H-Y et al. *JAMA Network Open.*

No	State	District	ADs	Composite score (%)		Decile		Districts requiring policy attention
				Score	SD	Score	SD	
366	Chhattisgarh	Korba	Yes	93.9	1.74	5	6	No
367	Chhattisgarh	Janjgir - champa	No	94	1.85	5	6	No
368	Chhattisgarh	Kabeerdham	No	96.2	1.2	9	2	No
369	Chhattisgarh	Rajnandgaon	Yes	95.4	1.41	8	3	No
370	Chhattisgarh	Mahasamund	Yes	94.1	1.47	5	4	No
371	Chhattisgarh	Dhamtari	No	94.9	1.6	7	5	No
372	Chhattisgarh	Uttar bastar kanker	Yes	93.2	1.42	4	4	No
373	Chhattisgarh	Narayanpur	Yes	93.7	1.81	5	6	No
374	Chhattisgarh	Bijapur	Yes	94.1	1.51	5	4	No
375	Madhya pradesh	Sheopur	No	92.5	2.15	3	8	Yes
376	Madhya pradesh	Morena	No	94.1	1.55	5	4	No
377	Madhya pradesh	Bhind	No	94.2	1.55	6	4	No
378	Madhya pradesh	Gwalior	No	93.9	1.74	5	6	No
379	Madhya pradesh	Datia	No	92.7	1.92	3	7	No
380	Madhya pradesh	Shivpuri	No	91.9	2.3	3	8	Yes
381	Madhya pradesh	Tikamgarh	No	94.2	1.71	5	5	No
382	Madhya pradesh	Chhatarpur	Yes	90.9	1.93	2	7	No
383	Madhya pradesh	Panna	No	89.8	2.28	2	8	Yes
384	Madhya pradesh	Sagar	No	90.4	2.44	2	9	Yes
385	Madhya pradesh	Damoh	Yes	91.3	2.24	2	8	Yes
386	Madhya pradesh	Satna	No	91.9	1.92	3	7	No
387	Madhya pradesh	Rewa	No	89.8	2.56	2	9	Yes
388	Madhya pradesh	Umaria	No	91.2	1.7	2	5	No
389	Madhya pradesh	Neemuch	No	95	1.46	7	4	No
390	Madhya pradesh	Mandsaur	No	94.5	1.59	6	5	No
391	Madhya pradesh	Ratlam	No	94	1.31	5	3	No
392	Madhya pradesh	Ujjain	No	93.6	2.14	4	8	No
393	Madhya pradesh	Dewas	No	94.2	1.6	6	5	No
394	Madhya pradesh	Dhar	No	94.1	1.76	5	6	No
395	Madhya pradesh	Indore	No	94.6	1.5	6	4	No
396	Madhya pradesh	Khargone (west nimar)	No	93.9	2	5	7	No

© 2022 Lee H-Y et al. *JAMA Network Open.*

No	State	District	ADs	Composite score (%)		Decile		Districts requiring policy attention
				Score	SD	Score	SD	
397	Madhya pradesh	Barwani	Yes	94.1	1.58	5	5	No
398	Madhya pradesh	Rajgarh	Yes	92.7	2.36	3	8	Yes
399	Madhya pradesh	Vidisha	Yes	94	1.71	5	5	No
400	Madhya pradesh	Bhopal	No	92.2	1.46	3	4	No
401	Madhya pradesh	Sehore	No	94.3	1.54	6	4	No
402	Madhya pradesh	Raisen	No	93	1.87	4	7	No
403	Madhya pradesh	Betul	No	93.7	1.95	4	7	No
404	Madhya pradesh	Harda	No	93.7	1.77	5	6	No
405	Madhya pradesh	Hoshangabad	No	92.6	1.86	3	6	No
406	Madhya pradesh	Katni	No	92.7	1.76	3	6	No
407	Madhya pradesh	Jabalpur	No	95	0.96	7	1	No
408	Madhya pradesh	Narsimhapur	No	94.7	1.47	6	4	No
409	Madhya pradesh	Dindori	No	92.6	1.87	3	7	No
410	Madhya pradesh	Mandla	No	93.1	1.81	4	6	No
411	Madhya pradesh	Chhindwara	No	94.5	1.41	6	3	No
412	Madhya pradesh	Seoni	No	94.8	1.29	7	3	No
413	Madhya pradesh	Balaghat	No	95.3	1.13	8	2	No
414	Madhya pradesh	Guna	Yes	92.8	2.21	4	8	No
415	Madhya pradesh	Ashoknagar	No	92.7	1.71	3	5	No
416	Madhya pradesh	Shahdol	No	93.9	1.64	5	5	No
417	Madhya pradesh	Anuppur	No	93.9	1.57	5	4	No
418	Madhya pradesh	Sidhi	No	90.2	1.82	2	6	No
419	Madhya pradesh	Singrauli	Yes	93.7	1.6	5	5	No
420	Madhya pradesh	Jhabua	No	93.8	1.55	5	4	No
421	Madhya pradesh	Alirajpur	No	94.8	1.65	7	5	No
422	Madhya pradesh	Khandwa (east nimar)	Yes	94.6	1.2	6	2	No
423	Madhya pradesh	Burhanpur	No	93.7	1.85	5	6	No
424	Gujarat	Kachchh	No	95.3	2.01	8	7	No
425	Gujarat	Banas kantha	No	94.9	1.44	7	4	No
426	Gujarat	Patan	No	95.8	1.11	8	2	No
427	Gujarat	Mahesana	No	92.3	2.23	3	8	Yes

© 2022 Lee H-Y et al. *JAMA Network Open.*

No	State	District	ADs	Composite score (%)		Decile		Districts requiring policy attention
				Score	SD	Score	SD	
428	Gujarat	Gandhinagar	No	94.6	1.55	6	4	No
429	Gujarat	Porbandar	No	95.3	1.14	8	2	No
430	Gujarat	Amreli	No	95.3	1.39	8	3	No
431	Gujarat	Anand	No	95.2	1.25	7	3	No
432	Gujarat	Dohad	Yes	94.8	1.79	7	6	No
433	Gujarat	Narmada	Yes	95.1	1.91	7	7	No
434	Gujarat	Bharuch	No	91.8	1.9	3	7	No
435	Gujarat	The dangs	No	96.2	1.24	9	2	No
436	Gujarat	Navsari	No	94.4	1.79	6	6	No
437	Gujarat	Valsad	No	96.4	1.23	9	2	No
438	Gujarat	Surat	No	95.5	1.77	8	6	No
439	Gujarat	Tapi	No	96.2	1.31	9	3	No
440	Dadra & nagar haveli and daman & diu	Diu	No	94	1.44	5	4	No
441	Dadra & nagar haveli and daman & diu	Daman	No	90.6	2.64	2	9	Yes
442	Dadra & nagar haveli and daman & diu	Dadra & nagar haveli and daman & diu nagar haveli	No	92.8	1.9	4	7	No
443	Maharashtra	Nandurbar	Yes	93.5	1.57	4	4	No
444	Maharashtra	Dhule	No	93.3	1.61	4	5	No
445	Maharashtra	Jalgaon	No	92.8	2.17	4	8	No
446	Maharashtra	Buldana	No	94.9	1.76	7	6	No
447	Maharashtra	Akola	No	94.1	1.87	5	7	No
448	Maharashtra	Washim	Yes	93.9	2.37	5	8	No
449	Maharashtra	Amravati	No	93.5	2.05	4	7	No
450	Maharashtra	Wardha	No	95.3	1.51	8	4	No
451	Maharashtra	Nagpur	No	95.2	1.73	7	6	No
452	Maharashtra	Bhandara	No	96.5	1.09	9	1	No
453	Maharashtra	Gondiya	No	95.9	1.28	9	3	No
454	Maharashtra	Gadchiroli	Yes	95.9	1.21	9	2	No

© 2022 Lee H-Y et al. *JAMA Network Open.*

No	State	District	ADs	Composite score (%)		Decile		Districts requiring policy attention
				Score	SD	Score	SD	
455	Maharashtra	Chandrapur	No	93.9	1.92	5	7	No
456	Maharashtra	Yavatmal	No	96.4	1.01	9	1	No
457	Maharashtra	Nanded	No	93.9	1.97	5	7	No
458	Maharashtra	Hingoli	No	94.6	1.69	6	5	No
459	Maharashtra	Parbhani	No	92.5	2.25	3	8	Yes
460	Maharashtra	Jalna	No	93.4	2.65	4	9	No
461	Maharashtra	Aurangabad	No	93	1.89	4	7	No
462	Maharashtra	Nashik	No	93.8	1.87	5	7	No
463	Maharashtra	Mumbai suburban	No	94	1.26	5	3	No
464	Maharashtra	Mumbai	No	94.2	1.14	5	2	No
465	Maharashtra	Raigarh	No	95	1	7	1	No
466	Maharashtra	Pune	No	93.6	2.02	4	7	No
467	Maharashtra	Ahmadnagar	No	95	0.92	7	1	No
468	Maharashtra	Bid	No	93.4	1.54	4	4	No
469	Maharashtra	Latur	No	95.9	0.81	9	1	No
470	Maharashtra	Osmanabad	Yes	96.2	0.96	9	1	No
471	Maharashtra	Solapur	No	94.2	1.38	6	3	No
472	Maharashtra	Satara	No	94.5	1.2	6	2	No
473	Maharashtra	Ratnagiri	No	95.5	1.19	8	2	No
474	Maharashtra	Sindhudurg	No	95.8	0.93	8	1	No
475	Maharashtra	Kolhapur	No	94.7	1.36	6	3	No
476	Maharashtra	Sangli	No	94.4	1.49	6	4	No
477	Andhra pradesh	Srikakulam	No	95.9	1.06	9	1	No
478	Andhra pradesh	Vizianagaram	Yes	95.9	1.21	9	2	No
479	Andhra pradesh	Visakhapatnam	Yes	96.4	1.18	9	2	No
480	Andhra pradesh	East godavari	No	95.8	1.3	9	3	No
481	Andhra pradesh	West godavari	No	95.4	1.12	8	2	No
482	Andhra pradesh	Krishna	No	94.7	1.22	7	2	No
483	Andhra pradesh	Guntur	No	96.5	1.17	9	2	No
484	Andhra pradesh	Prakasam	No	96.4	1.05	9	1	No
485	Andhra pradesh	Sri potti sriramulu nellore	No	94.5	1.27	6	3	No

© 2022 Lee H-Y et al. *JAMA Network Open.*

No	State	District	ADs	Composite score (%)		Decile		Districts requiring policy attention
				Score	SD	Score	SD	
486	Andhra pradesh	Y.s.r.	Yes	95.2	1.28	7	3	No
487	Andhra pradesh	Kurnool	No	96.1	1.35	9	3	No
488	Andhra pradesh	Anantapur	No	96.7	1.01	10	1	No
489	Andhra pradesh	Chittoor	No	96.4	1.19	9	2	No
490	Karnataka	Belgaum	No	95.7	1.48	8	4	No
491	Karnataka	Bagalkot	No	94.8	1.6	7	5	No
492	Karnataka	Bijapur	No	95.8	1.56	8	4	No
493	Karnataka	Bidar	No	93.6	1.8	4	6	No
494	Karnataka	Raichur	Yes	95.3	1.19	8	2	No
495	Karnataka	Koppal	No	94.3	1.78	6	6	No
496	Karnataka	Gadag	No	94.9	1.89	7	7	No
497	Karnataka	Dharwad	No	95.4	1.32	8	3	No
498	Karnataka	Uttara kannada	No	94.9	1.14	7	2	No
499	Karnataka	Haveri	No	95.9	1.09	9	1	No
500	Karnataka	Bellary	No	94.3	1.09	6	2	No
501	Karnataka	Chitradurga	No	95.3	1.53	8	4	No
502	Karnataka	Davanagere	No	94.8	1.26	7	3	No
503	Karnataka	Shimoga	No	94.4	1.88	6	7	No
504	Karnataka	Udupi	No	94.6	1.95	6	7	No
505	Karnataka	Chikmagalur	No	97	0.72	10	1	No
506	Karnataka	Tumkur	No	94.7	1.63	6	5	No
507	Karnataka	Bangalore	No	95.2	1.5	7	4	No
508	Karnataka	Mandya	No	96.3	1.16	9	2	No
509	Karnataka	Hassan	No	94.3	1.62	6	5	No
510	Karnataka	Dakshina kannada	No	94.4	2.4	6	9	No
511	Karnataka	Kodagu	No	95	1.46	7	4	No
512	Karnataka	Mysore	No	95.8	1.75	8	6	No
513	Karnataka	Chamarajanagar	No	95.4	1.46	8	4	No
514	Karnataka	Gulbarga	No	92.7	2.21	3	8	Yes
515	Karnataka	Yadgir	Yes	93.7	2.23	5	8	No
516	Karnataka	Kolar	No	95.1	1.4	7	3	No

© 2022 Lee H-Y et al. *JAMA Network Open.*

No	State	District	ADs	Composite score (%)		Decile		Districts requiring policy attention
				Score	SD	Score	SD	
517	Karnataka	Chikkaballapura	No	95.6	1.35	8	3	No
518	Karnataka	Bangalore rural	No	94.8	1.65	7	5	No
519	Karnataka	Ramanagara	No	94.6	1.84	6	6	No
520	Goa	North goa	No	96.6	1.19	9	2	No
521	Goa	South goa	No	96.9	1.2	10	2	No
522	Lakshadweep	Lakshadweep eep	No	98.2	0.81	10	1	No
523	Kerala	Kasaragod	No	98.4	0.78	10	1	No
524	Kerala	Kannur	No	97.3	1.27	10	3	No
525	Kerala	Wayanad	Yes	96.8	1.05	10	1	No
526	Kerala	Kozhikode	No	97.5	1.23	10	2	No
527	Kerala	Malappuram	No	97.5	1.39	10	3	No
528	Kerala	Palakkad	No	97	1.15	10	2	No
529	Kerala	Thrissur	No	96.9	0.89	10	1	No
530	Kerala	Ernakulam	No	97.6	0.82	10	1	No
531	Kerala	Idukki	No	97.9	0.69	10	1	No
532	Kerala	Kottayam	No	95.7	1.41	8	3	No
533	Kerala	Alappuzha	No	96.8	1.21	10	2	No
534	Kerala	Pathanamthitta	No	96.6	0.95	10	1	No
535	Kerala	Kollam	No	96.2	1.26	9	3	No
536	Kerala	Thiruvananthapuram	No	96.4	1.04	9	1	No
537	Tamil nadu	Thiruvallur	No	96.1	1.19	9	2	No
538	Tamil nadu	Chennai	No	98.1	0.7	10	1	No
539	Tamil nadu	Kancheepuram	No	96.5	1.01	9	1	No
540	Tamil nadu	Vellore	No	98.4	0.74	10	1	No
541	Tamil nadu	Tiruvannamalai	No	97.8	0.83	10	1	No
542	Tamil nadu	Viluppuram	No	97.2	1.06	10	1	No
543	Tamil nadu	Salem	No	97.7	0.78	10	1	No
544	Tamil nadu	Namakkal	No	96.2	1.37	9	3	No
545	Tamil nadu	Erode	No	98.1	0.77	10	1	No
546	Tamil nadu	The nilgiris	No	98	0.64	10	1	No
547	Tamil nadu	Dindigul	No	97.8	0.86	10	1	No

© 2022 Lee H-Y et al. *JAMA Network Open.*

No	State	District	ADs	Composite score (%)		Decile		Districts requiring policy attention
				Score	SD	Score	SD	
548	Tamil nadu	Karur	No	95.7	1.42	8	4	No
549	Tamil nadu	Tiruchirappalli	No	96.4	2.17	9	8	No
550	Tamil nadu	Perambalur	No	97.1	1.06	10	1	No
551	Tamil nadu	Ariyalur	No	97.4	1.1	10	2	No
552	Tamil nadu	Cuddalore	No	97.8	0.89	10	1	No
553	Tamil nadu	Nagapattinam	No	97	1.11	10	2	No
554	Tamil nadu	Thiruvarur	No	95.7	1.8	8	6	No
555	Tamil nadu	Thanjavur	No	96.6	1.42	10	3	No
556	Tamil nadu	Pudukkottai	No	95.9	1.36	9	3	No
557	Tamil nadu	Sivaganga	No	96.3	1.88	9	7	No
558	Tamil nadu	Madurai	No	97.1	1.01	10	1	No
559	Tamil nadu	Theni	No	97.8	1.15	10	2	No
560	Tamil nadu	Virudhunagar	Yes	96.3	1.11	9	2	No
561	Tamil nadu	Ramanathapuram	Yes	97.5	1.29	10	3	No
562	Tamil nadu	Thoothukkudi	No	96.6	1.39	10	3	No
563	Tamil nadu	Tirunelveli	No	97	1.08	10	1	No
564	Tamil nadu	Kanniyakumari	No	95.7	1.46	8	4	No
565	Tamil nadu	Dharmapuri	No	97.8	0.96	10	1	No
566	Tamil nadu	Krishnagiri	No	97.9	1.1	10	2	No
567	Tamil nadu	Coimbatore	No	97.5	0.89	10	1	No
568	Tamil nadu	Tiruppur	No	97.8	1.16	10	2	No
569	Puducherry	Yanam	No	96.5	1.1	9	2	No
570	Puducherry	Puducherryry	No	95.9	1.31	9	3	No
571	Puducherry	Mahe	No	98.3	0.39	10	1	No
572	Puducherry	Karaikal	No	97.4	1.04	10	1	No
573	Andaman & nicobar islands	Nicobars	No	96.4	0.97	9	1	No
574	Andaman & nicobar islands	North & middle andaman & nicobar islands	No	96	1.31	9	3	No
575	Andaman & nicobar islands	South andaman & nicobar islands	No	96.1	1.24	9	2	No

© 2022 Lee H-Y et al. *JAMA Network Open.*

No	State	District	ADs	Composite score (%)		Decile		Districts requiring policy attention
				Score	SD	Score	SD	
576	Arunachal pradesh	East siang	No	91	1.82	2	6	No
577	Arunachal pradesh	Kra daadi	No	88.5	4.18	1	10	Yes
578	Arunachal pradesh	Kurung kumey	No	93.8	1.34	5	3	No
579	Arunachal pradesh	Lohit	No	93.2	2.48	4	9	No
580	Arunachal pradesh	Longding	No	93.8	1.73	5	6	No
581	Arunachal pradesh	Namsai	Yes	95.2	1.63	7	5	No
582	Arunachal pradesh	Siang	No	91.2	1.99	2	7	No
583	Arunachal pradesh	Tirap	No	91.9	3.14	3	10	Yes
584	Arunachal pradesh	West siang	No	90.5	2.76	2	9	Yes
585	Assam	Biswanath	No	95.5	1.45	8	4	No
586	Assam	Charaideo	No	95.4	1.77	8	6	No
587	Assam	Dhubri	Yes	93.2	2.71	4	9	No
588	Assam	Hojai	No	95.7	1.56	8	4	No
589	Assam	Jorhat	No	95.9	1.57	9	4	No
590	Assam	Karbi anglong	No	95.3	1.83	8	6	No
591	Assam	Majuli	No	96.2	1.54	9	4	No
592	Assam	Nagaon	No	94.7	2.45	6	9	No
593	Assam	Sivasagar	No	95.6	1.99	8	7	No
594	Assam	Sonitpur	No	93.3	2.51	4	9	No
595	Assam	South salmara mancachar	No	94.8	2.03	7	7	No
596	Assam	West karbi anglong	No	93.9	1.93	5	7	No
597	Chhattisgarh	Balod	No	93.5	1.59	4	5	No
598	Chhattisgarh	Baloda bazar	No	92.2	1.93	3	7	No
599	Chhattisgarh	Balrampur	No	93.2	1.91	4	7	No
600	Chhattisgarh	Bastar	Yes	93.3	1.68	4	5	No
601	Chhattisgarh	Bemetara	No	96.1	1.09	9	2	No
602	Chhattisgarh	Bilaspur	No	93.1	1.51	4	4	No
603	Chhattisgarh	Dantewada	Yes	94.7	1.63	6	5	No
604	Chhattisgarh	Durg	No	96	1.1	9	2	No
605	Chhattisgarh	Gariyaband	No	94.2	2.06	6	8	No

© 2022 Lee H-Y et al. *JAMA Network Open.*

No	State	District	ADs	Composite score (%)		Decile		Districts requiring policy attention
				Score	SD	Score	SD	
606	Chhattisgarh	Kodagaon	Yes	94.6	1.43	6	4	No
607	Chhattisgarh	Mungeli	No	94.4	1.54	6	4	No
608	Chhattisgarh	Raipur	No	95.8	1.41	8	3	No
609	Chhattisgarh	Sukma	Yes	93	2.5	4	9	No
610	Chhattisgarh	Surajpur	No	94.5	1.92	6	7	No
611	Chhattisgarh	Surguja	No	94	2.01	5	7	No
612	Nct of delhi	Central	No	94.3	1.77	6	6	No
613	Nct of delhi	East	No	95.2	1.67	7	5	No
614	Nct of delhi	New delhi	No	94.3	1.51	6	4	No
615	Nct of delhi	North	No	92.4	2.33	3	8	Yes
616	Nct of delhi	North east	No	94.7	1.58	7	5	No
617	Nct of delhi	North west	No	95	1.2	7	2	No
618	Nct of delhi	Shahdara	No	94	1.68	5	5	No
619	Nct of delhi	South	No	93.8	1.71	5	5	No
620	Nct of delhi	South east	No	95.8	1.14	9	2	No
621	Nct of delhi	South west	No	94.9	1.54	7	4	No
622	Nct of delhi	West	No	94.4	1.41	6	3	No
623	Gujarat	Ahmadabad	No	95.3	1.2	8	2	No
624	Gujarat	Aravali	No	93.3	1.59	4	5	No
625	Gujarat	Bhavnagar	No	94.7	1.19	6	2	No
626	Gujarat	Botad	No	93.8	1.87	5	7	No
627	Gujarat	Chhota udaipur	No	93.7	1.87	5	7	No
628	Gujarat	Devbhumi dwarka	No	93.7	1.87	5	7	No
629	Gujarat	Gir somnath	No	95.2	1.24	7	2	No
630	Gujarat	Jamnagar	No	93.1	1.77	4	6	No
631	Gujarat	Junagadh	No	91.7	2.03	3	7	No
632	Gujarat	Kheda	No	92.1	1.86	3	7	No
633	Gujarat	Mahisagar	No	94	1.64	5	5	No
634	Gujarat	Morbi	No	94.7	1.61	6	5	No
635	Gujarat	Panch mahals	No	95.7	1.07	8	1	No
636	Gujarat	Rajkot	No	94.6	1.53	6	4	No

© 2022 Lee H-Y et al. *JAMA Network Open.*

No	State	District	ADs	Composite score (%)		Decile		Districts requiring policy attention
				Score	SD	Score	SD	
637	Gujarat	Sabar kantha	No	93.7	2.42	5	9	No
638	Gujarat	Surendranagar	No	92.6	2.19	3	8	Yes
639	Gujarat	Vadodara	No	93.7	1.8	4	6	No
640	Haryana	Bhiwani	No	93.7	1.76	5	6	No
641	Haryana	Charkhi dadri	No	95.4	1.34	8	3	No
642	Madhya pradesh	Agar malwa	No	94.5	1.77	6	6	No
643	Madhya pradesh	Shajapur	No	94.6	1.88	6	7	No
644	Maharashtra	Palghar	No	95	0.88	7	1	No
645	Maharashtra	Thane	No	95.2	1	7	1	No
646	Meghalay	East garo hills	No	92	2.34	3	8	Yes
647	Meghalay	East jantia hills	No	93.7	2.1	4	8	No
648	Meghalay	North garo hills	No	91	2.15	2	8	Yes
649	Meghalay	South west garo hills	No	92.3	2.9	3	10	Yes
650	Meghalay	South west khasi hills	No	93.3	1.22	4	2	No
651	Meghalay	West garo hills	No	92.1	2.32	3	8	Yes
652	Meghalay	West jaintia hills	No	93.3	2.32	4	8	No
653	Meghalay	West khasi hills	No	93	2.01	4	7	No
654	Punjab	Fazilka	No	95.6	1.02	8	1	No
655	Punjab	Firozpur	Yes	94.7	1.78	6	6	No
656	Punjab	Gurdaspur	No	94.8	1.66	7	5	No
657	Punjab	Pathankot	No	93.9	1.81	5	6	No
658	Telangana	Adilabad	No	95.2	1.4	7	3	No
659	Telangana	Bhadradri kothagudem	Yes	95.5	1.78	8	6	No
660	Telangana	Hyderabad	No	96.6	0.99	10	1	No
661	Telangana	Jagitial	No	94.2	2.39	6	8	No
662	Telangana	Jangoan	No	95.2	1.06	7	1	No
663	Telangana	Jayashankar bhupalapally	No	94.7	1.59	6	5	No
664	Telangana	Jogulamba gadwal	No	96.2	1.15	9	2	No
665	Telangana	Kamareddy	No	95.8	1.55	8	4	No
666	Telangana	Karimnagar	No	94.3	1.34	6	3	No

© 2022 Lee H-Y et al. *JAMA Network Open.*

No	State	District	ADs	Composite score (%)		Decile		Districts requiring policy attention
				Score	SD	Score	SD	
667	Telangana	Khammam	No	96.3	1.07	9	1	No
668	Telangana	Komaram bheem asifabad	Yes	95.9	1.27	9	3	No
669	Telangana	Mahabubabad	No	95.3	1.16	8	2	No
670	Telangana	Mahabubnagar	No	96	1.78	9	6	No
671	Telangana	Mancherial	No	94.7	1.48	6	4	No
672	Telangana	Medak	No	93.6	1.94	4	7	No
673	Telangana	Medchal-malkajgiri	No	95.3	1.79	8	6	No
674	Telangana	Nagarkurnool	No	95.2	1.45	7	4	No
675	Telangana	Nalgonda	No	96.6	0.97	10	1	No
676	Telangana	Nirmal	No	94.3	1.32	6	3	No
677	Telangana	Nizamabad	No	94	1.5	5	4	No
678	Telangana	Peddapalli	No	94	1.31	5	3	No
679	Telangana	Rajanna sircilla	No	95.2	1.34	7	3	No
680	Telangana	Ranga reddy	No	95.3	1.42	8	3	No
681	Telangana	Sangareddy	No	95.5	1.16	8	2	No
682	Telangana	Siddipet	No	95.1	1.37	7	3	No
683	Telangana	Suryapet	No	94.6	1.83	6	6	No
684	Telangana	Vikarabad	No	93.9	2.23	5	8	No
685	Telangana	Wanaparthy	No	96.6	1.18	9	2	No
686	Telangana	Warangal rural	No	94.7	1.14	6	2	No
687	Telangana	Warangal urban	No	95.7	1.34	8	3	No
688	Telangana	Yadadri bhuvanagiri	No	95.9	1.08	9	1	No
689	Tripura	Gomati	No	91.1	1.39	2	3	No
690	Tripura	Khowai	No	89.6	2.37	2	8	Yes
691	Tripura	North tripura	No	94.4	1.34	6	3	No
692	Tripura	Sepahijala	No	91.1	1.53	2	4	No
693	Tripura	South tripura	No	88.1	2.58	1	9	Yes
694	Tripura	Unakoti	No	85.7	3.07	1	10	Yes
695	Tripura	West tripura	No	92.7	2.12	4	8	No
696	Uttar pradesh	Amethi	No	87.4	3.83	1	10	Yes
697	Uttar pradesh	Budaun	No	89	3.35	1	10	Yes

© 2022 Lee H-Y et al. *JAMA Network Open.*

No	State	District	ADs	Composite score (%)		Decile		Districts requiring policy attention
				Score	SD	Score	SD	
698	Uttar pradesh	Ghaziabad	No	92.2	1.92	3	7	No
699	Uttar pradesh	Hapur	No	91.7	2.34	3	8	Yes
700	Uttar pradesh	Moradabad	No	89.3	2.09	2	8	Yes
701	Uttar pradesh	Muzaffarnagar	No	91.7	1.67	3	5	No
702	Uttar pradesh	Rae bareli	No	90.2	2.41	2	9	Yes
703	Uttar pradesh	Sambhal	No	87.5	4.37	1	10	Yes
704	Uttar pradesh	Shamli	No	91.6	2.35	3	8	Yes
705	Uttar pradesh	Sultanpur	No	89.5	2.54	2	9	Yes
706	West bengal	Paschim barddhaman	No	94.9	1.17	7	2	No
707	West bengal	Purba barddhaman	No	94.2	1.79	6	6	No

AD: aspirational district / SD: Standard deviation

© 2022 Lee H-Y et al. *JAMA Network Open.*
